# Cyprus Women’s Health Research (COHERE) initiative: determining the relative burden of women’s health conditions and related co-morbidities in an Eastern Mediterranean population

**DOI:** 10.1186/s12905-019-0750-1

**Published:** 2019-04-03

**Authors:** M. B. Hocaoglu, S. Gurkas, T. Karaderi, B. Taneri, K. Erguler, B. Barin, E. M. Bilgin, G. Eralp, M. Allison, N. Findikli, K. Boynukalin, M. Bahceci, H. Naci, K. Vincent, S. A. Missmer, C. M. Becker, K. T. Zondervan, N. Rahmioglu

**Affiliations:** 1 Department of Palliative Care, Policy and Rehabilitation, Cicely Saunders Institute, King’s College London, London, UK; 2 Faculty of Medicine and Faculty of Arts and Sciences, Department of Psychology, Eastern Mediterranean University, Famagusta, Northern Cyprus; 3 Istanbul Faculty of Medicine, Istanbul University, Istanbul, Turkey; 4 DTU Health Technology, Technical University of Denmark, 2800 Lyngby, Denmark; 5 Novo Nordisk Foundation Center for Protein Research, Faculty of Health and Medical Sciences, University of Copenhagen, 2200 Copenhagen, Denmark; 6 Faculty of Arts and Sciences, Department of Biological Sciences, Eastern Mediterranean University, Famagusta, Northern Cyprus; 7 Wellcome Centre for Human Genetics, University of Oxford, Oxford, UK; 8 Institute for Public Health Genomics (IPHG), Department of Genetics and Cell Biology, Research Institute GROW, Faculty of Health, Medicine & Life Sciences, University of Maastricht, Maastricht, The Netherlands; 9 Cyprus Women’s Health Research Society (CoHERS), Nicosia, Northern Cyprus; 10 EMMES Corporation, Rockville, MD USA; 11 Bahceci IVF Hospital Cyprus, Bahceci Health Group, Nicosia, Northern Cyprus; 12 Gunes Women’s Health Clinic, Nicosia, Northern Cyprus; 13 Jinomer Women’s Health Clinic, Kyrenia, Northern Cyprus; 14 Bahceci Health Group, Istanbul, Turkey; 15 London School of Economics and Political Science, London, UK; 16 Oxford Endometriosis CaRe Centre, Nuffield Department of Women’s and Reproductive Health, University of Oxford, Oxford, UK; 17 Department of Epidemiology, Harvard T.H. Chan School of Public Health, Boston, MA USA; 18 Department of Obstetrics, Gynecology and Reproductive Biology, College of Human Medicine, Michigan State University, Grand Rapids, MI USA

**Keywords:** Benign women’s health conditions, Prevalence, Endometriosis, Uterine fibroids, PCOS, Chronic pain conditions, Eastern Mediterranean population

## Abstract

**Background:**

There is lack of population level data on prevalence and distribution of common benign women’s health conditions such as endometriosis, uterine fibroids, polycystic ovary syndrome from the Eastern Mediterranean region despite their significant consequences on quality of life. In particular, there is complete absence of any health statistics from Northern Cyprus, which is an emerging region in Europe. The Cyprus Women’s Health Research (COHERE) Initiative is the first large-scale cross-sectional study in the region, aiming to determine the relative burden of benign women’s health conditions and related co-morbidities in women living in Northern Cyprus.

**Methods:**

The COHERE Initiative is a cross-sectional study aiming to recruit 8000 women aged 18–55 years and residing for at least the past 5 years in Northern Cyprus. The study is composed of two main steps: (1) Baseline recruitment, including (i) completion of a detailed health questionnaire, which is an expanded version of the World Endometriosis Research Foundation (WERF) Endometriosis Phenome Harmonisation Project (EPHect) standardised questionnaire, including questions on demographics, menstrual history, hormone use, pregnancy, pain (pelvic pain, bladder and bowel pain, migraine), medical history, family history of illnesses, medication use, life-style factors in relation to a wide range of reproductive and endocrine conditions, resource use (ii) measurement of weight, height, waist/hip circumference and blood pressure, (iii) collection of saliva samples for genotyping. (2) Gynaecology clinic follow up, including a pelvic ultrasound scan (USS). There is also a follow-up food frequency questionnaire (FFQ) targeted to all women taking part in the baseline recruitment with an aim to collect more detailed data on dietary habits.

**Discussion:**

The COHERE Initiative will generate prevalence rates for conditions, define the clinical profiles for women’s health conditions, and estimate the economic burden of these conditions in Northern Cyprus. The results will also provide insights into the current status of health-care among women living in a currently under-investigated region. The genetic findings will inform future gene mapping studies for investigation of the heritable component of conditions in this population/region. Moreover, the results will be compared with other centres collecting data using EPHect tools globally and will help determine population differences and similarities in disease patterns and clinical profiles. The COHERE Initiative will serve as a resource to conduct hypothesis-driven follow-up studies investigating effect of the ‘Mediterranean life-style’ as well as genetic factors on common benign women’s health conditions that maybe specific to Eastern Mediterranean populations.

## Background

Cyprus is the third largest Mediterranean island with approximately 300,000 Turkish Cypriot and 700,000 Greek Cypriot residents. Due to the, to date, unresolved political circumstances, Northern Cyprus, has been relatively isolated from the rest of Europe for more than 45 years [1]. Although Cyprus has been a member of the European Union (EU) since May 1, 2004, the acquis communautaire is suspended in the northern part of the island [2], and unfortunately official collaborations between the north and south administrations and institutions have been absent. Consequently, population-level health data from Northern Cyprus have not been included in health statistics reported for Cyprus [3]. Moreover, there is an absence of population-level data on common benign women’s health conditions such as endometriosis, uterine fibroids, polycystic ovary syndrome (PCOS) and related co-morbidities generally from the Eastern Mediterranean region. It is well established that women’s cohorts such as the Nurses’ Health Study from the USA [4] and the Million Women Study from the UK [5] have been crucial in investigating how various reproductive and lifestyle factors affect women’s health. Hence, establishment of a resource to investigate women’s health conditions and causal environmental and genetic factors that can be specific to populations is necessary for the Eastern Mediterranean populations.

The Cyprus Women’s Health Research (COHERE) Initiative aims to establish a women’s health cohort in Cyprus, with current emphasis on the north for the above-mentioned reasons of data paucity to collect vital health, morbidity, and resource use data, and investigate factors affecting women’s health and care seeking. COHERE Initiative is a population-based, cross-sectional study, based on a household/workplace sampling method utilising an extended version of the Endometriosis Phenome and Biobanking Harmonisation Project (EPHect) questionnaire [6–11] that consists of validated instruments used in previous studies, targeting 10% of all women aged 18–55 (*N* = 8000) living in Northern Cyprus. Study participants completing the questionnaire also have the opportunity to provide a saliva sample for genotyping to understand the underlying genetic architecture of this population. Moreover, participants are invited to a clinical follow-up visit at a women’s health clinic that includes a transvaginal or transabdominal pelvic ultrasound scan (USS) that provides clinical data on diagnosis of uterine fibroids, polycystic ovaries, and some endometriosis cases.

This study will provide the first systematically collected population health data for Northern Cyprus - an emerging region in Europe for which public health issues have been unexplored to date. The study will aid the understanding of regional women’s health and illness patterns, and the personal, social and economic burden of symptomatology and disease. Disease rates, clinical profiles, and healthcare statistics of women in this population will be utilised to assess the relative burden of disease, and results will form the basis for targeted hypothesis-driven follow-up studies. Moreover, the Cypriot adaption of the ‘Mediterranean lifestyle’ allows for investigation of the influence of both environmental as well as genetic factors specific to Eastern Mediterranean populations. With this study, the genetic architecture of the population will be unravelled to better inform future gene association studies, investigating genetic risk variants for disease/traits from this population. Moreover, given the genetic susceptibilities, it will lay the foundation to promote changes in potential environmental modifiers for common complex women’s health conditions in the region. Furthermore, the health statistics that will be generated from the study will inform the health authorities about prevalence and distribution of certain women’s health issues that can be used in development of data-driven health strategies in the region.

## Method/design

### Study aims

This study aims to (1) estimate prevalence rates of gynaecological conditions and associated symptomatology, auto-immune, inflammatory, metabolic and pain co-morbidity profiles; (2) investigate how various reproductive and lifestyle factors affect women’s health including diet, exercise, employment patterns, oral contraceptive use, childbirth and breastfeeding, family history of illness, in relation to a wide range of reproductive and endocrine conditions, (3) investigate the geospatial distribution of identified conditions in Northern Cyprus and also comparison of disease rates with other 19 centres collecting data using EPHECT based data collection tool, (4) understand the genetic architecture of this population by genotyping DNA extracted from saliva samples collected from a minimum of 1000 participating women, (5) quantify women’s access to health care and estimate the economic burden of diseases such as endometriosis in Northern Cyprus, (6) gain insights into women’s perceptions of research and interest in participation in subsequent follow-up studies.

### Ethics

The study was approved by the Oxford Tropical Research Ethics Committee (OxTREC) of the University of Oxford (OxTREC reference: 37–17). The study also received local ethics approval from the Eastern Mediterranean University Ethics Committee (ETK00-2017-0240).

### Eligibility

Women aged 18 to 55 years, who are either citizens of Northern Cyprus or have been residing in Northern Cyprus for the last 5 years, and who are able to give informed consent are eligible to participate in the study. Women younger than 18 or aged over 55, non-citizen women who have been residing in Northern Cyprus for less than 5 years, women who cannot understand the information on the participant information sheet or informed consent form due to being very unwell or illiterate will be excluded from the study.

### Recruitment procedures

**Fig. 1:**
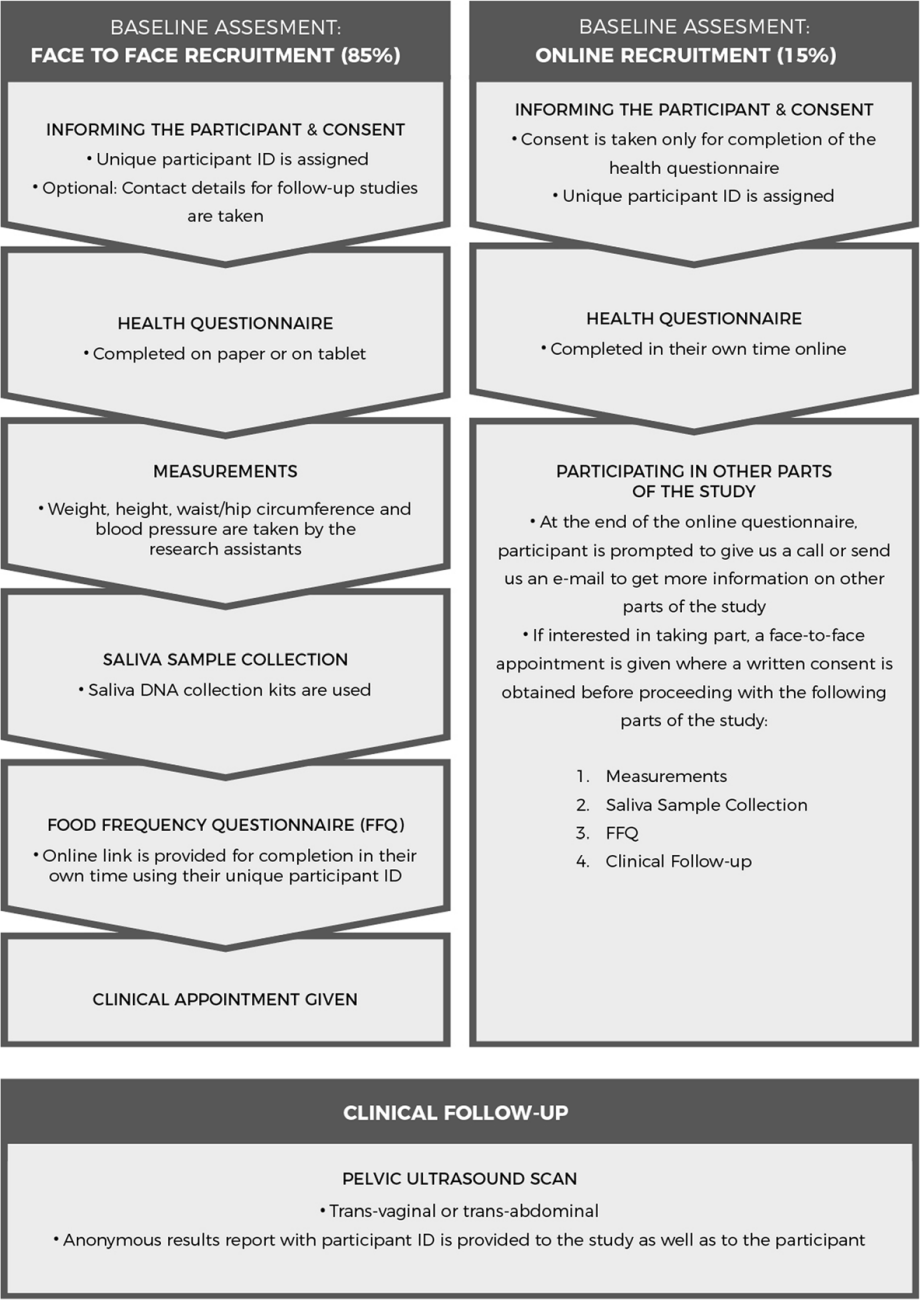
Overview of the study design

Participants are recruited through two different routes (Fig. 1): (i) Face-to-face recruitment where household/workplace visits are conducted by the research assistants to inform the women about the study and invite them to participate in the study. If women are interested in taking part, they provide informed consent and then complete the questionnaire. Additionally, they have the option of providing a saliva sample and/or undergoing an appointment at the women’s health clinic for a pelvic USS. (ii) Online recruitment, which is promoted through dedicated social media (https://web.facebook.com/KISAAInisiyatifi) page and study page (http://www.cohereinitiative.com). Participants can anonymously complete the study questionnaire online. At the beginning of the questionnaire, they read the participant information sheet and complete an online consent. At the end of the online questionnaire, if they would like the opportunity to take part in the clinical follow-up or provide saliva samples, they are instructed to call the research assistants, using their assigned study participant codes from the online questionnaire. The research assistants arrange an appointment to meet the participant face-to-face to complete a written consent followed by saliva sample collection and/or scheduling of the women’s health clinic appointment.

### Study design

COHERE Initiative is a population-based cross-sectional study composed of two main steps (Fig. 1): (i) Baseline assessment includes informing participant, getting consent, collecting of questionnaire-based health and lifestyle data, taking anthropometric measurements, collecting saliva sample for DNA extraction and genotyping, (ii) Clinical follow-up includes a pelvic USS by an experienced gynaecologist.

#### Baseline assessment

All women are informed about the study and the consenting women are given a unique participant ID to be used throughout the study. Participants are asked to complete the baseline EPHect-based health questionnaire [10] with validated instruments used in previous studies either on paper or on tablet computer (Additional file 1). The questionnaire takes around 20–30 min. After the completion of the baseline health EPHect-based questionnaire, basic anthropometric measurements are taken from consenting women. A saliva sample is collected for DNA extraction from a subset of the consenting women. As the last item on the baseline assessment, women are provided with the food frequency questionnaire (FFQ) [12–14], which they can complete in their own time. There are two options to complete the FFQ: (1) Online, using their unique participant ID, they can login to complete FFQ with the given questionnaire web-link, (2) Paper-based, on which the research assistants put a sticker with the unique participant ID and hand the questionnaire to the participant to complete in their own time and drop their FFQ to the near-by questionnaire collection boxes. Clinical appointments for the consenting participants are given by the research assistant at the end of the baseline assessment at most within 2 weeks of their baseline assessment.

#### Clinical follow-up

At the clinical follow-up, participants undergo a pelvic USS performed by an experienced gynaecologist. Ideally, this is a transvaginal procedure but can be transabdominal if preferred by the participant. As well as being linked to the unique participant ID, scan reports are also provided to the participant in the form of a medical report.

### Data collection tools

#### Expanded EPHect-based health questionnaire

Each consenting woman is asked to complete the EPHect-based health questionnaire. EPHect consists of a set of tools to standardise globally the collection of clinical/lifestyle data and samples across studies to allow for more effective large-scale international collaborative research studies on endometriosis. We have utilized the EPHect health questionnaire as the basis of this study questionnaire and expanded it to capture data on other benign women’s health conditions such as uterine fibroids, polycystic ovary syndrome, various chronic pain conditions such as bladder pain and migraine, endocrine conditions such as thyroid conditions. The questionnaire was translated into Turkish, and then back-translated into English. Differences between the original English version and translated English version were compared by an expert panel. The Turkish version was revised where there were differences. The Turkish version was piloted in 100 Turkish Cypriot women of different age segments (Ages: 18–30; 30–40; 40–55) and education levels (Highest education attained: primary school diploma; high school diploma; university degree). Comments from the participants for any unclear questions were also incorporated to the final version. For the Turkish validated and published versions of the questionnaire, scales were identified and incorporated into the questionnaire including the SF36 v2 [15] and Pain Catastrophizing Scale [16].

#### Measurements

Research assistants take blood pressure, weight, height, waist and hip circumference measurements. Each measurement is taken twice; where there is a significant difference between the two measurements, a third measurement is also taken.

#### Saliva collection kits

Saliva samples are collected for DNA extraction for genotyping [17–19]. A total of 2 ml of saliva sample is collected by the participant actively spitting into an ORAGENE-saliva collection tube. The standard operation protocol that comes with the saliva collection kit is followed by each participant and overseen by the research assistant. This is a risk-free, non-invasive method for high-quality DNA extraction from saliva samples.

#### Food frequency questionnaire

The last item on the baseline assessment is to provide women with the Food-Frequency Questionnaire (FFQ), which they can complete in their own time. The semi-quantitative food frequency questionnaire that has been validated for use in Turkish adults [12] is utilised for this cohort of women, and has also been used in a global study previously [13, 14].

#### Pelvic ultrasound scan (USS)

Either a transvaginal or transabdominal pelvic USS is conducted on consenting women at the clinical follow-up to examine their reproductive organs. The pelvic USS results provide clinical data on diagnosis of uterine fibroids, polycystic ovaries and some of the endometriosis cases. Both transvaginal and transabdominal USS are safe, risk-free tests routinely conducted in gynaecology clinics. Pelvic USS uses high-frequency sound waves (no radiation) to create images of the female reproductive organs and the pelvic cavity. Some participants may find transvaginal scans uncomfortable since it involves the transducer being inserted into the vagina and this would be inappropriate for women who are *virgo intacta*. Therefore, as an alternative, we are offering a transabdominal USS where the transducer moves only over the abdomen to image the pelvis.

### Statistical analysis

Data analysis will commence after the data collection period (January 31, 2018-January 31, 2020) ends. Descriptive statistics including characterisation of the women participating in the study will be provided where the prevalence rates (and 95% confidence intervals) for self-reported and clinically validated medical conditions will be estimated. Association of life-style factors and various symptomatology between determined disease case groups and healthy controls will be investigated using the statistical computing software package R.

The open-source geographic information system platform QGIS (Open Source Geospatial Foundation) will be used to visualise data. Spatial and/or temporal clustering among the investigating conditions and symptomatology will be explored and Geographically Weighted Regression (GWR) analysis [20] will be performed to investigate hidden relationships among the conditions and symptomatology with regards to geospatial distribution.

Quality control of the genetic data will be conducted using PLINK software [21] followed by cluster analysis such as principal component analysis, implemented in R, to identify individuals with ancestral differences that will aid future studies aiming to investigate the genetic background of conditions/traits [22]. Moreover, genetic association analysis, where large enough sample sets are present, using linear and logistic regression modelling for quantitative and qualitative traits respectively, will be conducted comparing the differences in frequency and distribution of genetic variants.

Furthermore, the economic burden of disease will be estimated using direct and indirect costs.

### Power calculations

We are aiming to recruit a random ~ 10% sample of the 82,104 women between the ages of 18–55 residing in the 5 main regions of in Northern Cyprus including Lefkosa, Girne, Gazimagusa, Guzelyurt and Iskele following the geographic population densities. The aimed total sample size of 8000 women will allow us to detect prevalence rates as low as 0.5% in the overall population (40 cases: 7960 controls). Moreover, it will also allow us to estimate prevalence rates as low as 1% in the most populated cities such as Lefkosa and Gazimagusa (target recruitment of 2716 and 1981 women respectively), and in comparison, in the less populated regions such as Guzelyurt (target recruitment of 808 women) a prevalence rate of 2% and in smaller rural villages, prevalence rates of 5–10% will be estimable. Please see the Additional file 2: Power calculations for details of the sample size calculations and recruitment targets for each region. Moreover, where we have the needed case numbers, we aim to look for association between lifestyle factors and conditions. For example, the overall sample size of 8000 allows for detection of 2–16% increase in exposure rates in cases compared to controls, where disease prevalence rates are 1–50% and exposure rates in controls are 10–90%.

### Data management

All study data will be entered on a custom-made cloud-based survey platform maintained by our study technology funder DMD consulting with servers employing secure cloud computing environment located in Frankfurt, Germany. The cloud-based data are securely transferred to high-compliance University of Oxford servers on a monthly basis. Direct access will be granted to authorised representatives from the University of Oxford and any host institution for monitoring and/or audit of the study to ensure compliance with regulations.

The participants will be identified by a unique participant ID number in the database and nowhere in the electronic database, patient identifiable data are to be stored. Paper records (including consent forms) will be held in locked cabinets in the Chief Investigator’s office at Eastern Mediterranean University during data collection. After data collection is finished, these will be transferred to University of Oxford premises and stored in a locked cabinet at the University of Oxford until 31st January, 2028 (end date of the study) and archived for another 10 years after the completion of the study.

## Discussion

This study is the first population-based study that will collect data in a standardized way allowing for investigation of women health conditions, related co-morbidities and symptomatology from an Eastern Mediterranean population. The unique setting of the target Turkish Cypriot population will also provide critical insights into the successes and disparities of health-care among women living in an internationally under-represented community that may be applicable across the globe.

The standardised data collection tools utilised in the study will allow for comparison of disease rates, clinical profiles, and healthcare statistics of women in this population with at least 19 other EPHect centres globally (https://endometriosisfoundation.org/ephect/) to better understand the relative burden of disease.

Moreover, the results of this study will form the basis for targeted, hypothesis-driven follow-up studies. They will facilitate addressing of the environmental factors, such as diet as well as the genetic factors causal for women’s health conditions that may be specific to Eastern Mediterranean populations. The genetic architecture of women in Northern Cyprus will be revealed to better inform future genetic association studies.

This study is envisaged to promote evidence-based reproductive medicine in the region, not only benefitting the local population but also providing a basis for an Eastern Mediterranean women’s health resource.

## Additional files

Additional file 1: Baseline study questionnaire. (PDF 710 kb)

Additional file 2: Power calculations. (DOCX 15 kb)

## Abbreviations

COHERE: Initiative Cyprus Women’s Health Research Initiative
EPHect: Endometriosis Phenome Harmonisation Project
FFQ: Food frequency questionnaire
GWR: Geographically Weighted Regression
OxTREC: Oxford Tropical Research Ethics Committee
PCOS: Polycystic ovary syndrome
QGIS: Open Source Geospatial Foundation
SF36 v2: 36- Item Short Form Survey Version 2
USS: Ultrasound scan
WERF: World Endometriosis Research Foundation
