# Supplementary material for: Cyprus Women’s Health Research (COHERE) initiative: determining the relative burden of women’s health conditions and related co-morbidities in an Eastern Mediterranean population
Source: BMC Womens Health. 2019 Apr 3;19:50. doi: 10.1186/s12905-019-0750-1 (PMC6446287; doi:10.1186/s12905-019-0750-1)
Supplement: Supplementary file 1 — Baseline study questionnaire. (PDF 710 kb) [file 12905_2019_750_MOESM1_ESM.pdf]

Cyprus Women's Health Research Initiative Questionnaire

Overall Health

The following questions ask for your views about your health and how you feel about life in general. If you are unsure about how to answer any question, try and think about your overall health and give the best answer you can. Do not spend too much time answering, as your immediate response is likely to be the most accurate.

A1. In general, would you say your health is:

- ☐ Excellent
- ☐ Very good
- ☐ Good
- ☐ Fair
- ☐ Poor

A2. Compared to one year ago, how would you rate your health in general now?

- ☐ Much better than one year ago
- ☐ Somewhat better than one year ago
- ☐ About the same
- ☐ Somewhat worse now than one year ago
- ☐ Much worse now than one year ago

A3. The following questions are about activities you might do during a typical day.
Does your health limit you in these activities?
If so, how much?

	**Yes, limited a lot**	**Yes, limited a little**	**No, not limited at all**
(a) Vigorous activities, such as running, lifting heavy objects, participating in strenuous sports	☐	☐	☐
(b) Moderate activities, such as moving a table, pushing a vacuum, bowling or playing golf	☐	☐	☐
(c) Lifting or carrying groceries	☐	☐	☐
(d) Climbing several flights of stairs	☐	☐	☐
(e) Climbing one flight of stairs	☐	☐	☐
(f) Bending, kneeling or stooping	☐	☐	☐
(g) Walking more than a mile	☐	☐	☐
(h) Walking half a mile	☐	☐	☐
(i) Walking 100 yards	☐	☐	☐
(j) Bathing and dressing yourself	☐	☐	☐

A4. During the past 4 weeks, how much of the time have you had any of the following problems with your work or other regular daily activities as a result of your physical health?

	All of the time	Most of the time	Some of the time	A little of the time	None of the time
(a) Cut down on the amount of time you spent on work and other activities	☐	☐	☐	☐	☐
(b) Accomplished less than you would like	☐	☐	☐	☐	☐
(c) Were limited in the kind of work or other activities	☐	☐	☐	☐	☐
(d) Had difficulty performing the work or other activities (e.g. it took more effort)	☐	☐	☐	☐	☐

A5. During the past 4 weeks, how much of the time have you had any of the following problems with your work or other regular daily activities as a result of any emotional problems (such as feeling depressed or anxious)?

	All of the time	Most of the time	Some of the time	A little of the time	None of the time
(a) Cut down on the amount of time you spent on work and other activities	☐	☐	☐	☐	☐
(b) Accomplished less than you would like	☐	☐	☐	☐	☐
(c) Did work or other activities less carefully than usual	☐	☐	☐	☐	☐

A6. During the past 4 weeks, to what extent have your physical health or emotional problems interfered with your normal social activities with family, neighbours or groups?

☐ Not at all
☐ Slightly
☐ Moderately
☐ Quite a bit
☐ Extremely

A7. How much bodily pain have you had during the past 4 weeks?

☐ None
☐ Very mild
☐ Mild
☐ Moderate
☐ Severe
☐ Very severe

A8. During the past 4 weeks, how much did pain interfere with your normal work (including both outside the home and housework)?

☐ Not at all
☐ Slightly
☐ Moderately
☐ Quite a bit
☐ Extremely

A9. These questions are about how you feel and how things have been with you during the **past 4 weeks**. For each question, please give one answer that comes closest to the way you have been feeling.

	All of the time	Most of the time	Some of the time	A little of the time	None of the time
(a) Did you feel full of life?	☐	☐	☐	☐	☐
(b) Have you been very nervous?	☐	☐	☐	☐	☐
(c) Have you felt so down in the dumps that nothing would cheer you up?	☐	☐	☐	☐	☐
(d) Have you felt calm and peaceful?	☐	☐	☐	☐	☐
(e) Did you have a lot of energy?	☐	☐	☐	☐	☐
(f) Have you felt downhearted and low?	☐	☐	☐	☐	☐
(g) Did you feel worn out?	☐	☐	☐	☐	☐
(h) Have you been happy?	☐	☐	☐	☐	☐
(i) Did you feel tired?	☐	☐	☐	☐	☐

A10. During the **past 4 weeks**, how much of the time has your **physical health** or **emotional problems** interfered with your social activities (like visiting friends, relatives, etc.)?

☐ All of the time
☐ Most of the time
☐ Some of the time
☐ A little of the time
☐ None of the time

A11. How TRUE or FALSE is each of the following statements for you?

	Definitely true	Mostly true	Not sure	Mostly false	Definitely false
(a) I seem to get ill more easily than other people	☐	☐	☐	☐	☐
(b) I am as healthy as anybody I know	☐	☐	☐	☐	☐
(c) I expect my health to get worse	☐	☐	☐	☐	☐
(d) My health is excellent	☐	☐	☐	☐	☐

Menstrual history and hormones

B1. How old were you when you had your first menstrual period?

☐ 8 years or younger ☐ 11 ☐ 14 ☐ 17 years or older
☐ 9 ☐ 12 ☐ 15 ☐ uncertain
☐ 10 ☐ 13 ☐ 16

B2. Have you had any periods **in the last 3 months**? *(We mean bleeding for which you needed a tampon or sanitary pad, NOT discharge (spotting) for which you needed a panty liner only)*

☐ No → **continue with question B2.1**
☐ Yes → **Skip to question B2.4**

If you have NOT had periods in the last 3 months:

B2.1. What was the reason for not having periods?

☐ Taking hormones continuously (*e.g. the Pill, injections, Mirena, HRT*)
☐ Pregnant/breastfeeding
☐ Menopause (stopped having periods) → **If yes**: B2.1.1. Which of the following symptoms apply to you at this time? (Please ✓ the appropriate box for each symptom)

Symptoms	None	Mild	Moderate	Severe	Very Severe
(a) Hot flushes, sweating (episodes of sweating)	☐	☐	☐	☐	☐
(b) Heart discomfort (unusual awareness of heart beat, heart skipping, heart racing, tightness)	☐	☐	☐	☐	☐
(c) Sleep problems (difficulty in falling asleep, difficulty in sleeping through, waking up early)	☐	☐	☐	☐	☐
(d) Depressive mood (feeling down, sad, on the verge of tears, lack of drive, mood swings)	☐	☐	☐	☐	☐
(e) Irritability (feeling nervous, inner tension, feeling aggressive)	☐	☐	☐	☐	☐
(f) Anxiety (inner restlessness, feeling panicky).	☐	☐	☐	☐	☐
(g) Physical and mental exhaustion (general decrease in performance, impaired memory, decrease in concentration, forgetfulness)	☐	☐	☐	☐	☐
(h) Sexual problems (change in sexual desire, in sexual activity and satisfaction)	☐	☐	☐	☐	☐
(i) Bladder problems (difficulty in urinating, increased need to urinate, bladder incontinence)	☐	☐	☐	☐	☐
(j) Dryness of vagina (sensation of dryness or burning in the vagina, difficulty with sexual intercourse)	☐	☐	☐	☐	☐
(k) Joint and muscular discomfort (pain in the joints, rheumatoid complaints)	☐	☐	☐	☐	☐

☐ Unsure
☐ Other (*Please describe*) ____________________

B2.2. Approximately how many periods have you had **over the last 12 months**? _____

B2.3. When was your last period?

☐ 3-6 months ☐ 7-12 months ☐ Over 12 months

→ **Please now continue with question B3.**

If you have had periods in the last 3 months, please answer the following questions about your recent periods.

B2.4. Were your periods in the last 3 months natural or hormone-induced (*e.g. on the Pill, injections, Mirena or HRT*)?

☐ Natural ☐ Hormone induced

B2.5. When was the first day of your last menstrual period (LMP)?

LMP___/___/_______ ☐ Uncertain
DD MM YYYY

B2.6. Were your periods in the last 3 months regular? (expected date – every 28 days?)

☐ extremely regular (period starts 1-2 days before or after it is expected)
☐ very regular (period starts 3-4 days before or after it is expected)
☐ regular (period starts 5-7 days before or after it is expected)
☐ somewhat irregular (period starts 8-20 days before or after it is expected)
☐ irregular (period starts more than 20 days before or after it is expected)

B2.7. How many days of bleeding did you usually have each period in the last 3 months? *(Not counting discharge/spotting for which you need a panty liner only)*

_____ days or ☐ Too irregular to say

B2.8. The figure below shows examples of the amount of bleeding you can experience **every four hours** during your period. Please describe the amount of bleeding you typically experience four-hourly during your period **at its heaviest**, and **on average**.

At its heaviest?

☐ Spotting
☐ Light
☐ Moderate
☐ Heavy

On average?

☐ Spotting
☐ Light
☐ Moderate
☐ Heavy

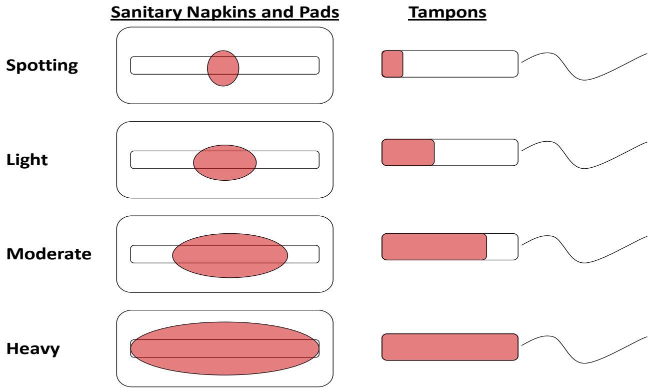

B2.9. In the last 3 months, how many days were there between the first day of one period and the first day of the next **on average**? *(Not including spotting)*

☐ < 24 days
☐ 24-31 days
☐ 32-38 days
☐ 39-50 days
☐ 51+ days
☐ Too irregular to estimate

B3. Please list below all hormones you have **ever** used for any reason (acne, bad cramping, irregular periods, birth control, fertility treatments). For each hormone used, please indicate what type of hormone it was using the number indicated for the categories below. Please also tell us the age you first used them and total time of use. If you cannot remember the name of the hormone you used, please write "unknown" in the first column. **If you have never used hormones** before, please tick ☐ and proceed to **B5**.

1=Combined birth control pill (e.g. Marvelon, Yasmin, Microgynon)
2=Progestin only birth control pill ("mini-pill", e.g. Cerazette, Micronor)
3=Unsure of which type of oral birth control pill
4=Progestin injection/shot (e.g. Depo provera)
5=Transdermals: patches (e.g. OrthoEvra, Climara), dots (Vivelle dot)
6=Vaginal ring (NuvaRing)
7=Progesterone containing coil/IUD (Mirena)
8=Hormonalimplant (Implanon/Nexplanon)
9=Oral progestins to regulate the cycle (e.g. medroxyprogesterone acetate [Provera], dydrogesterone [Duphaston],dienogest [Visanne], Norethisterone)
10=GnRH agonist injection/shot (e.g.leuprolilide (leuproline) acetate [Prostap], goserelin [Zoladex])
11=Norethindrone acetate (Aygestin)
12=Danazol (please specify if used vaginally or orally)
13=Hormone replacement therapy (e.g. Premarin, Provera)

14=Other
15=Don't know what type of hormone

Name of hormone	Type of hormone (Please enter the number associated with the category above.)	Age started	Used within the last 3 months?	Total time used
For example: Yasmin	*1*	*18*	✔ *No* ☐ *Yes*	*…… months 2 years*
1.	....	....	☐ No ☐ Yes	…… months …… years
2.	....	....	☐ No ☐ Yes	…… months …… years
3.	....	....	☐ No ☐ Yes	…… months …… years
4.	....	....	☐ No ☐ Yes	…… months …… years
5.	....	....	☐ No ☐ Yes	…… months …… years

B4. What are/were your reasons for using hormones? *(Please tick ✓ all that apply)*

☐ Birth control / pregnancy prevention
☐ Irregular periods
☐ Heavy periods
☐ Acne
☐ Polycystic ovarian syndrome (PCOS)
☐ Ovarian cyst
☐ Pelvic pain or pain with periods
If yes: B4.1. Did hormones help with the pain? ☐ Yes ☐ No
B4.2. Did you ever discontinue or change hormones because they were not effective enough at controlling pain? ☐ Yes ☐ No
☐ Other (Please specify): ____

B5. Have you ever used a non-hormonal coil/IUD?

☐ No
☐ Yes → **If yes: B5.1.** At what age did you first use a non-hormonal coil/IUD? ____

B6. Have you ever used emergency contraception?

☐ No ☐ Yes → **If yes: B6.1.** How many times have you used emergency contraception? ______

Medical Screening and Resource Use

C1. Do you need a carer to assist you with your daily activities? (for basic hygiene, to help you move, administration of drugs, performing treatments, etc.)

☐ No → **Skip to C2**

☐ Yes → **If yes:** C1.1. Who is your main carer?

☐ Family member

☐ Another non-contracted person (e.g. friend)

☐ Professional carer → **If yes:**

C1.1.1. How many hours of care do you receive a week? ____

C1.1.2. What is the cost per hour/day? ____

C1.1.3. Who pays for the service and how much? ____

C2.1. At what age did you first visit a gynaecologist? ____ years old.

C2.2. Do you visit a gynaecologist at least once a year?

☐ No ☐ Yes

C3. Have you ever had a pap-smear test?

☐ No → **Skip to C4**

☐ Yes → **If yes: C3.1**. At what age did you have your first pap-smear test? ____ years old

C3.2. How often do you have a pap-smear test?

☐ Once a year

☐ Every other year

☐ Every three years

☐ Every five years

☐ Not regularly

C4. Have you had the Human Papillomavirus (HPV) vaccination?

☐ No ☐ Yes

C5. Do you self-exam your breasts for lumps, changes or discharge regularly?

☐ No ☐ Yes

C6. Have you ever had a clinical breast screening (ultra-sound, mammogram, MRI)?

☐ No → **Skip to C7**

☐ Yes → **If yes: C6.1.** How old were you when you had the first clinical breast screening?

____ years old

C6.2. How often do you get clinical breast screening (ultra-sound, mammogram, MRI)?

☐ Once a year

☐ Every other year

☐ Every three years

☐ Every five years

☐ Not regularly

C7. Have you had the below medical tests or screenings (prescribed by a doctor) in the last 6 months?

	**Please ✓all the apply**	**How was this funded?**
Blood Tests	☐	☐ State insurance ☐ Private insurance ☐ Out-of pocket
Ultra-sound (e.g. vaginal, thyroid, abdominal, breast)	☐	☐ State insurance ☐ Private insurance ☐ Out-of pocket
MRI/CT Scan	☐	☐ State insurance ☐ Private insurance ☐ Out-of pocket
Other (*Please Specify*)________	☐	☐ State insurance ☐ Private insurance ☐ Out-of pocket

C8. How many visits to specialists have you had to undergo in the last 6 months? Please list them below.

**Specialist**	**Number of Visits**
1.________________________	____________________
2.________________________	____________________
3.________________________	____________________
4.________________________	____________________
5_________________________	____________________

C9. How many visits to the emergency service have you made in the last 6 months?

_____ ☐ By myself or ☐ Ambulance

C10. Have you been admitted to hospital in the last 12 months?

☐ No → **Skip to D1**

☐ Yes→ **If yes:**

C10.1. How many times have you been admitted to hospital in the last 12 months? ____ times

C10.2. How many days have you spent as an inpatient in the hospital in the last 12 months? ___ days

C10.3. During the hospital admissions in the last 12 months, have you undergone any procedures?

☐ No → **Skip to C10.4**

☐ Yes→ **If yes:** Please list them below:

1. ____
2. ____
3. ____

C10.4. Please rate how satisfied you were with the health care received using a scale from 0 to 10 where 0= Not satisfied at all and 10= Very satisfied.

**Not satisfied at all**									**Very satisfied**	
0	1	2	3	4	5	6	7	8	9	10

C11. How many times have you used the following means of transport in the last 6 months for disease related travel to the health clinic, hospital, etc.?

☐ Private car: ____ times
☐ Bus: ____ times
☐ Aeroplane (if visiting another country) ____ times
☐ Ambulance ____ times

Pregnancy and fertility

D1. Have you ever been pregnant (confirmed by a positive pregnancy test, including miscarriages, ectopic pregnancies or terminations)?

☐ No → **Skip to D3**
☐ Yes, please complete the table below.

	**Pregnancy**							
	**1st**	**2nd**	**3rd**	**4th**	**5th**	**6th**	**7th**	**8th**
**How old were you at the start of the pregnancy?**								
(Please write your age at each pregnancy)	....	....	....	....	....	....	....	....
**What fertility treatment was used, if any, for this pregnancy?**								
Natural conception: no fertility treatment	☐	☐	☐	☐	☐	☐	☐	☐
Fertility drugs by pills to stimulate ovulation (clomid, clomiphene)	☐	☐	☐	☐	☐	☐	☐	☐
Intrauterine insemination (IUI)	☐	☐	☐	☐	☐	☐	☐	☐
In vitro fertilization (IVF/ICSI)	☐	☐	☐	☐	☐	☐	☐	☐
**What was the outcome of this pregnancy?** ***(Please tick ✓ all that apply)***								
Single live birth	☐	☐	☐	☐	☐	☐	☐	☐
Twins or triplets	☐	☐	☐	☐	☐	☐	☐	☐
Miscarriage	☐	☐	☐	☐	☐	☐	☐	☐
Stillbirth	☐	☐	☐	☐	☐	☐	☐	☐
Termination (abortion)	☐	☐	☐	☐	☐	☐	☐	☐
Tubal or pregnancy in other location outside the uterus	☐	☐	☐	☐	☐	☐	☐	☐
Molar	☐	☐	☐	☐	☐	☐	☐	☐
Currently pregnant	☐	☐	☐	☐	☐	☐	☐	☐
**How many weeks were you pregnant?**								
Less than 24 weeks	....	....	....	....	....	....	....	....
24-28 weeks	....	....	....	....	....	....	....	....
29-32 weeks	....	....	....	....	....	....	....	....
33-36 weeks	....	....	....	....	....	....	....	....
37 or more weeks	....	....	....	....	....	....	....	....
**If this pregnancy was a miscarriage, tubal/ectopic, or if you had a termination, how was this managed?**								
Surgically (D&C, ERPC)	☐	☐	☐	☐	☐	☐	☐	☐
Medically (using tablets, orally and/or vaginally)	☐	☐	☐	☐	☐	☐	☐	☐
No management was needed	☐	☐	☐	☐	☐	☐	☐	☐
**If this pregnancy resulted in a birth, was the delivery vaginal or via Caesarean section?**								
Vaginal birth	☐	☐	☐	☐	☐	☐	☐	☐
Caesarean section	☐	☐	☐	☐	☐	☐	☐	☐
**Did you go into labour and if so, was it induced or did it begin on its own?**								
No labour	☐	☐	☐	☐	☐	☐	☐	☐
Spontaneous labour	☐	☐	☐	☐	☐	☐	☐	☐
Induced labour	☐	☐	☐	☐	☐	☐	☐	☐
**Did you have any of the following complications related to pregnancy or breast feeding?**								
Gestational diabetes	☐	☐	☐	☐	☐	☐	☐	☐
Pregnancy-related high blood pressure	☐	☐	☐	☐	☐	☐	☐	☐
Pre-eclampsia Toxemia	☐	☐	☐	☐	☐	☐	☐	☐
Mastitis/breast infection	☐	☐	☐	☐	☐	☐	☐	☐
HELLP syndrome	☐	☐	☐	☐	☐	☐	☐	☐
Hyperemesis gravidarum	☐	☐	☐	☐	☐	☐	☐	☐
Pre-term birth (birth before 37 weeks)	☐	☐	☐	☐	☐	☐	☐	☐
Other:	☐	☐	☐	☐	☐	☐	☐	☐
Other:	☐	☐	☐	☐	☐	☐	☐	☐

D1. (continued)

	Pregnancy 1st	2nd	3rd	4th	5th	6th	7th	8th
**If this pregnancy resulted in a birth, for how long did you breastfeed?**								
(Please write the number of months you breastfed or write 0 if you did not breastfeed.);if you breastfed for less than 1 month, please write'1'	....	....	....	....	....	....	....	....
**What was the birth weight and gender of the baby?**								
Weight in kg	....	....	....	....	....	....	....	....
Height in cm	....	....	....	....	....	....	....	....
Girl	....	....	....	....	....	....	....	....
Boy	....	....	....	....	....	....	....	....
**If more than one baby please continue below:**								
Weight in kg	....	....	....	....	....	....	....	....
Height in cm	....	....	....	....	....	....	....	....
Girl	....	....	....	....	....	....	....	....
Boy	....	....	....	....	....	....	....	....

D2. After the birth of your **last** child and **after you stopped breastfeeding**, how were your menstrual cycles different compared to before you became pregnant with your first child? (*Please tick ✓all that apply*)

☐ No change
☐ Periods more regular
☐ Periods less regular
☐ Periods more painful
☐ Periods less painful

D3. Have you ever tried to get pregnant for more than 6 months in a row without succeeding?

☐ No → Skip to E1
☐ Yes → **If yes:** D3.1. What was the longest amount of time that you tried, whether or not you actually got pregnant? _____ months

D4. Have you or your partner ever had any tests/investigations to find out why you were not getting pregnant?

☐ No
☐ Yes → **If yes:** D4.1. What were the results of these tests? (*Please tick ✓all that apply*)

☐ Endometriosis (Chocolate cyst)
☐ Adhesions
☐ Blocked tubes
☐ Polycystic ovary syndrome (PCOS)
☐ Pelvic inflammatory disease
☐ No/irregular ovulation
☐ Poor sperm count/quality
☐ Uterine fibroids
☐ No cause was found
☐ I can't remember
☐ Other……………………

D5. Did you ever seek treatment for infertility in any clinic?

☐ No

☐ Yes → **If yes: D5.1.** Please tell us about any fertility treatment you have used.

	**Never used**	**Used within the last three months**	**Used, but not within the last three months**	**Number of cycles (if applicable)**
Intercourse timed specifically to conceive	☐	☐	☐	……..
Fertility-focused intercourse	☐	☐	☐	……..
Fertility drugs by pills to stimulate ovulation (clomid, clomiphene or any other drug in pill form)	☐	☐	☐	……..
Fertility drugs by Injection (gonadotropins, HCG, or any other drug by injection)	☐	☐	☐	……..
Progesterone (vaginal or intramuscular injection)	☐	☐	☐	……..
Insemination with your partner's semen	☐	☐	☐	……..
Intrauterine insemination with a donor's semen	☐	☐	☐	……..
In vitro fertilization (IVF)	☐	☐	☐	……..
In vitro fertilization with intracytoplasmic sperm injection (ICSI)	☐	☐	☐	……..
In vitro fertilization with eggs from a donor	☐	☐	☐	……..

D6. If you ever had **IVF, ICSI, or IVF with donor egg(s):** After what step did your IVF cycle(s) end?

(Please tick ✓ all that apply)

☐ Ovarian stimulation (did not have eggs retrieved)

☐ Egg retrieval (did not have embryos transferred)

☐ Embryo transfer (did not have a positive pregnancy test)

☐ Chemical pregnancy (had a positive pregnancy test but no heartbeat on ultrasound)

☐ Clinical pregnancy (heartbeat detected, but had a pregnancy loss before the end of 12 weeks)

☐ Pregnancy loss or stillbirth after 12 weeks

☐ Live birth

Pain

E1. Everyone experiences painful situations at some point in their lives. Such experiences may include headaches, tooth pain, joint, back or muscle pain. People are often exposed to situations that may cause pain such as illness, injury, dental procedures, or surgery.

We are interested in the types of thoughts and feelings that you have when you are in pain. Listed below are thirteen statements describing different thoughts and feelings that may be associated with pain. Using the scale, please indicate the degree to which you have these thoughts and feelings when you are experiencing pain.

	Not at all	To a slight degree	To a moderate degree	To a great degree	All the time
I worry all the time about whether the pain will end	☐	☐	☐	☐	☐
I feel I can't go on	☐	☐	☐	☐	☐
It's terrible and I think it's never going to get any better	☐	☐	☐	☐	☐
It's awful and I feel that it overwhelms me	☐	☐	☐	☐	☐
I feel I can't stand it anymore	☐	☐	☐	☐	☐
I become afraid that the pain will get worse	☐	☐	☐	☐	☐
I keep thinking of other painful events	☐	☐	☐	☐	☐
I anxiously want the pain to go away	☐	☐	☐	☐	☐
I can't seem to keep it out of my mind	☐	☐	☐	☐	☐
I keep thinking about how much it hurts	☐	☐	☐	☐	☐
I keep thinking about how badly I want the pain to stop	☐	☐	☐	☐	☐
There's nothing I can do to reduce the intensity of the pain	☐	☐	☐	☐	☐
I wonder whether something serious may happen	☐	☐	☐	☐	☐

The following questions ask about pelvic pain <u>with</u> your periods *(including irregular bleeding or bleeding while on hormonal treatments, but not spotting).*

By 'pelvic pain' we mean any type of pain (cramping, shooting, stabbing, etc.) in the lower part of your belly, as shown by the shaded area in this picture:

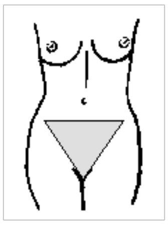

E2. Have you **ever** had pain during your periods?

☐ No pain
☐ Mild cramps (medication never or rarely needed)
☐ Moderate cramps (medication usually needed)
☐ Severe cramps (medication and bed rest needed)

→ **E2.1.** At what age did you start having period pain? ___ years

If you have had a period in the last 3 months, please complete the following questions, otherwise, please tick here ___ and continue to question E12.

E3. How much pain did you have **during your last period**?

☐ No pain → **Skip to question E10**
☐ Mild cramps (medication never or rarely needed)
☐ Moderate cramps (medication usually needed)
☐ Severe cramps (medication and bed rest needed)

E4. Did you take any pain-killers for period pain **during your last period**? (*Please tick ✓ all that apply*)

☐ No
☐ Yes, pain-killers that were prescribed by a doctor
☐ Yes, pain-killers bought over the counter without prescription (e.g. aspirin, ibuprofen, paracetamol/acetaminophen [e.g. panadol], naproxen [e.g. apranax])?

E5. Did you take hormones to help alleviate menstrual pain **during your last period** and if so, did it help to alleviate your pain?

☐ Did not take hormones for pain
☐ Yes, but pain was not alleviated
☐ Yes, pain was at least somewhat alleviated

E6. During your last period, did your period pain prevent you from going to work or school or carrying out your daily activities (even if taking pain-killers)?

☐ No ☐ Yes

E7. During your last period, did you have to lie down for any part of the day or longer because of your period pain?

☐ No ☐ Yes

E8. Please rate how severe your period pain was at its worst **during your last period** using a scale from 0 to 10 where 0=no pain and 10=worst imaginable pain.

**No pain**										**Worst imaginable pain**
0	1	2	3	4	5	6	7	8	9	10

E9. The following questions are about your bowel movements/stool **when you had period pain in the last 3 months.**

When you had period pain in the last 3 months, how often…	**Never/ Rarely**	**Some-times**	**Often**	**Most of the time**	**Always**
(a) …did this pain get better or stop after you had a bowel movement?	☐	☐	☐	☐	☐
(b) …did this pain get worse after you had a bowel movement?	☐	☐	☐	☐	☐
(c) …did you have more frequent bowel movements when the pain started?	☐	☐	☐	☐	☐
(d) …did you have less frequent bowel movements when the pain started?	☐	☐	☐	☐	☐
(e) …were your stools looser when the pain started?	☐	☐	☐	☐	☐
(f) …were your stools harder when the pain started?	☐	☐	☐	☐	☐

E10. In the last 12 months, how often have you had period pain?

☐ Never
☐ Occasionally (less than a quarter of my periods)
☐ Often (a quarter to half of my periods)
☐ Usually (more than half of my periods)
☐ Always (every period)

E11. Please rate how severe your period pain was at its worst **in the last 12 months** using a scale from 0 to 10 where 0=no pain and 10=worst imaginable pain.

**No pain**										**Worst imaginable pain**
0	1	2	3	4	5	6	7	8	9	10

The following questions are about the time in your life when your period pain was at its worst.

E12. How old were you when your period pain was at its worst? ………… years

E13. Please rate how severe your period pain was when it was at its worst using a scale from 0 to 10 where 0=no pain and 10=worst imaginable pain.

**No pain**										**Worst imaginable pain**
0	1	2	3	4	5	6	7	8	9	10

E14. During the time in your life when your period pain was at its worst, were you taking any medication to help alleviate the pain? (*Please tick ✓all that apply*)

☐ No
☐ Yes, pain-killers that were prescribed by a doctor
☐ Yes, pain-killers bought over the counter without prescription (e.g. aspirin, ibuprofen, paracetamol/acetaminophen, naproxen)?
☐ Yes, hormones, but pain was not alleviated
☐ Yes, hormones, pain was at least somewhat alleviated

The following questions are about pain during or after vaginal intercourse or penetration.
By 'pelvic pain' we mean any type of pain (cramping, shooting, stabbing, etc.) in the lower part of your belly, as shown by the shaded area in this picture:

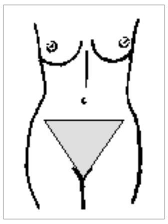

We remind you that any information you give will be treated in complete confidence.

***If however you do not wish to answer these questions, please tick here ___ and skip to question* E27.**

If you have never had intercourse, please check here ___ and skip to question E27

E15. Have you ever had pain during intercourse or in the 24 hours following vaginal sexual intercourse/penetration**?**

- ☐ No → Please skip to question **E27**
- ☐ Yes → **If yes**: E15.1. At what age did this pain start? ____

E16. When did you last have vaginal intercourse?

- ☐ In the last month
- ☐ 1-3 months ago
- ☐ 4-12 months ago
- ☐ More than 12 months ago → **If so: E16.1.** Did you avoid intercourse because of pelvic pain?
  ☐ No ☐ Yes

If you had vaginal intercourse more than 12 months ago, please go to question E27

E17. When you last had vaginal intercourse/penetration, did you have pelvic pain during or in the 24 hours following sexual intercourse**?**

- ☐ No → if No, please continue with question E27
- ☐ Yes, during intercourse/penetration
- ☐ Yes, in the 24 hours following intercourse/penetration
- ☐ Yes, both during intercourse/penetration and in the 24 hours following

E18. When you last had vaginal intercourse/penetration, where did you feel the pain? *(Please tick ✓ all that apply)*

- ☐ At the entrance of the vagina
- ☐ Deep inside the vagina
- ☐ In the abdomen/pelvis
- ☐ Other location → **If yes**: E18.1. Please describe: ____

E19. Please rate how severe your pain was at its worst **during the last time you had vaginal intercourse/penetration** using a scale from 0 to 10 where 0=no pain and 10=worst imaginable pain.

**No pain**										**Worst imaginable pain**
0	1	2	3	4	5	6	7	8	9	10

E20. Please rate how severe your pain was at its worst **in the 24 hours after the last time you had vaginal intercourse/penetration** using a scale from 0 to 10 where 0=no pain and 10=worst imaginable pain.

**No pain**										**Worst imaginable pain**
0	1	2	3	4	5	6	7	8	9	10

E21. During times you had vaginal intercourse/penetration **in the last 12 months**, how often did you have pelvic pain during or in the 24 hours after intercourse?

- ☐ Never
- ☐ Occasionally (less than a quarter of times)
- ☐ Often (a quarter to half of the times)
- ☐ Usually (more than half of the times)
- ☐ Always (every time)

E22. In the last 12 months, was there a time of the month in which vaginal intercourse/penetration was more painful than at other times? *(Please tick ✓ all that apply)*

	Was intercourse/vaginal penetration attempted during this time frame?		**If yes**, was it more painful at this time than other times?
**E22.1.** During a period?	☐ No ☐ Yes	→	☐ No ☐ Yes
**E22.2.** A few days before a period	☐ No ☐ Yes	→	☐ No ☐ Yes
**E22.3.** A few days after a period	☐ No ☐ Yes	→	☐ No ☐ Yes
**E22.4.** At mid cycle (around ovulation)	☐ No ☐ Yes	→	☐ No ☐ Yes

E23. In the last 12 months, did you ever **interrupt** vaginal intercourse/penetration because of pelvic pain?

☐ No

☐ Yes

E24. In the last 12 months, did you ever **avoid** vaginal intercourse/penetration because of pelvic pain?

☐ No

☐ Yes

The following questions are about the time in your life when your pain with vaginal intercourse/penetration was <u>at its worst</u>.

E25. How old were you when your pain with vaginal intercourse/penetration was at its worst? .... years

E26. Please rate how severe your pain with vaginal intercourse/penetration was when it was <u>at its worst</u> using a scale from 0 to 10 where 0=no pain and 10=worst imaginable pain.

**No pain**										**Worst imaginable pain**
0	1	2	3	4	5	6	7	8	9	10

The questions in this section ask about <u>pelvic/lower abdominal pain in general</u>.

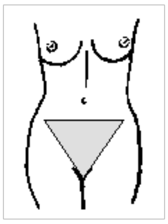

By 'pelvic pain' we mean any type of pain (cramping, shooting, stabbing, etc.) in the lower part of your belly, as shown by the shaded area in this picture:

*Please **<u>do not count</u>**: pain related to periods or intercourse, pregnancy or childbirth, any surgery, sports-related or other injury, food poisoning or stomach flu.*

E27. Have you ever experienced pelvic pain in general? ***<u>Do not count</u>**: pain caused by menstrual cramps, intercourse, surgery, pregnancy, childbirth, sports-related or other injury, food poisoning, or stomach flu.*

☐ No → **Skip to E46**

☐ Yes → **E27.1** At what age did you start having this pelvic pain? ___ years

E27.2 When did you last have this pain?

☐ In the last month

☐ 1-3 months ago

☐ 4-6 months ago

☐ 7-12 months ago → Please go to question **E41**

☐ longer than 12 months ago

(4-6 months ago, 7-12 months ago, longer than 12 months ago → Please go to question **E41**)

E28. To what extent has your pain interfered with your normal social activities with each of the following activities **in the last 3 months**:

	**Not At All**	**Slightly**	**Moderately**	**Quite A Bit**	**Extremely**	**Not Applicable**
Work or school:	☐	☐	☐	☐	☐	☐
Daily activities at home:	☐	☐	☐	☐	☐	☐
Sleep:	☐	☐	☐	☐	☐	☐
Sexual intercourse:	☐	☐	☐	☐	☐	☐
Exercise/sports:	☐	☐	☐	☐	☐	☐
Social activities	☐	☐	☐	☐	☐	☐

E29. Approximately how long in total did you have this pain for **in the last 3 months**?

☐ Less than one day ☐ One day a week
☐ One day ☐ More than one day a week
☐ Two to three days ☐ Every day

E30. Do you usually have this pain at about the same time in your cycle? *(Please tick ✓ all that apply)*

☐ No
☐ Yes: a few days before a period
☐ Yes: a few days after a period
☐ Yes: at mid cycle (around ovulation)
☐ Yes, other……………………………………….

E31. Have you taken any medication to help alleviate this pain **in the last 3 months**? *(Please tick ✓ all that apply)*

☐ No
☐ Yes, pain-killers that were prescribed by a doctor
☐ Yes, pain-killers bought over the counter without prescription (e.g. aspirin, ibuprofen,paracetamol/acetaminophen, naproxen)
☐ Yes, hormones, but pain was not alleviated
☐ Yes, hormones, pain was at least somewhat alleviated

E32. Please rate how severe your pelvic pain was **<u>at its worst</u> in the last 3 months** using a scale from 0 to 10 where 0=no pain and 10=worst imaginable pain.

**No pain**										**Worst imaginable pain**
0	1	2	3	4	5	6	7	8	9	10

E33. Please rate how severe your pelvic pain was **<u>on average</u> in the last 3 months** using a scale from 0 to 10 where 0=no pain and 10=worst imaginable pain.

**No pain**										**Worst imaginable pain**
0	1	2	3	4	5	6	7	8	9	10

E34. Please rate how severe your pelvic pain was **<u>at its worst</u>, during your last internal gynaecological examination,** going from no pain (0) to worst possible pain (10)**:**

**No pain**										**Worst imaginable pain**
0	1	2	3	4	5	6	7	8	9	10

E35. When you had pelvic pain **in the last 3 months**, what did it feel like?

Throbbing	☐ None	☐ Mild	☐ Moderate	☐ Severe
Shooting	☐ None	☐ Mild	☐ Moderate	☐ Severe
Stabbing	☐ None	☐ Mild	☐ Moderate	☐ Severe
Sharp	☐ None	☐ Mild	☐ Moderate	☐ Severe
Cramping	☐ None	☐ Mild	☐ Moderate	☐ Severe
Gnawing	☐ None	☐ Mild	☐ Moderate	☐ Severe
Hot-Burning	☐ None	☐ Mild	☐ Moderate	☐ Severe
Aching	☐ None	☐ Mild	☐ Moderate	☐ Severe
Heavy	☐ None	☐ Mild	☐ Moderate	☐ Severe
Tender	☐ None	☐ Mild	☐ Moderate	☐ Severe
Splitting	☐ None	☐ Mild	☐ Moderate	☐ Severe
Tiring-Exhausting	☐ None	☐ Mild	☐ Moderate	☐ Severe
Sickening	☐ None	☐ Mild	☐ Moderate	☐ Severe
Fearful	☐ None	☐ Mild	☐ Moderate	☐ Severe
Punishing-Cruel	☐ None	☐ Mild	☐ Moderate	☐ Severe

E36. What makes your pelvic pain worse? (*Please tick ✓all that apply*)

☐ Sitting	☐ Stress	☐ Standing or walking
☐ Full bladder or urinating	☐ Time of day	☐ Exercise
☐ Bowel movement	☐ Full meal	☐Coughing/sneezing
☐ Nothing makes my pain worse	☐ Weather	☐ Constipation
☐ Intercourse or orgasm	☐ Exercise	☐ Other, please specify__________________________

E37. What helps your pelvic pain? (*Please tick ✓all that apply*)

☐ Pain medication	☐ Hot bath
☐ Relaxation	☐ Meditation
☐ Lying down	☐ Laxatives / enema
☐ Music	☐ TENS Unit
☐ Massage	☐ Emptying bladder
☐ Ice	☐ Hot water bottle
☐ Heating pad	☐ Nothing helps
☐ Bowel movement	☐ Other: ....

E38. The following questions are about your bowel movements/stool when you had pelvic pain **in the last 3 months:**

When you had pelvic pain in the last 3 months, how often…	**Never/ Rarely**	**Some-times**	**Often**	**Most of the time**	**Always**
(a) …did this pain get better or stop after you had a bowel movement?	☐	☐	☐	☐	☐
(b) …did this pain get worse after you had a bowel movement?	☐	☐	☐	☐	☐
(c) …did you have more frequent bowel movements when the pain started?	☐	☐	☐	☐	☐
(d) …did you have less frequent bowel movements when the pain started?	☐	☐	☐	☐	☐
(e) …were your stools looser when the pain started?	☐	☐	☐	☐	☐
(f) ...were your stools harder when the pain started?	☐	☐	☐	☐	☐

The following questions are about the time <u>in your life</u> when your pelvic/lower abdominal pain was <u>at its worst</u>. *Please **do not count**: pain related to periods or intercourse, pregnancy or childbirth, any surgery, sports-related or other injury, food poisoning or stomach flu.*

E39. How old were you when your pelvic/lower abdominal pain was **at its worst**? ……………. years old

E40. Please rate how severe your pelvic/lower abdominal pain was when it was at its worst using a scale from 0 to 10 where 0=no pain and 10=worst imaginable pain.

No pain										Worst imaginable pain
0	1	2	3	4	5	6	7	8	9	10

E41. During the time in your life when your pelvic/lower abdominal pain was at its worst were you taking any medication to help alleviate the pain? (*Please tick ✓all that apply*)

- ☐ No
- ☐ Yes, pain-killers that were prescribed by a doctor
- ☐ Yes, pain-killers bought over the counter without prescription (e.g. aspirin, ibuprofen, paracetamol/acetaminophen, naproxen)
- ☐ Yes, hormones, but pain was not alleviated
- ☐ Yes, hormones, pain was at least somewhat alleviated

E42. Have you ever received a diagnosis for the pain from a doctor?

- ☐ No
- ☐ Yes (*Please tick ✓all that apply*):
  - ☐ Irritable Bowel Syndrome
  - ☐ Inflammatory bowel disease (e.g. Crohn's or Ulcerative Colitis)
  - ☐ Endometriosis
  - ☐ Fibroid(s)
  - ☐ Ovarian cyst
  - ☐ Pelvic inflammatory disease/infection
  - ☐ Painful bladder/interstitial cystitis (NOT a bacterial bladder infection)
  - ☐ Stress
  - ☐ Other: (*Please describe*) ………………………………………………….

E43. The following questions are about **bladder pain in the last 7 days.**

In the past 7 days.....	**Never**	**Rarely**	**Often**	**Most of the time**	**Always**
(a) .... when you urinated, how often was it because of pain in your bladder?	☐	☐	☐	☐	☐
(b) ..... how often did you still feel the need to urinate just after you urinated?	☐	☐	☐	☐	☐
(c) ..... how often did you urinate to avoid pain in your bladder from getting worse?	☐	☐	☐	☐	☐
(d) ..... how often did you have a feeling of pressure in your bladder?	☐	☐	☐	☐	☐
(e) ..... how often did you have pain in your bladder?	☐	☐	☐	☐	☐
(f) ..... how bothered were you by frequent urination during the daytime?	☐	☐	☐	☐	☐
(g) ..... how bothered were you by having to get up during the night to urinate?	☐	☐	☐	☐	☐

E44. Please rate on the following scale **your worst bladder pain** in the last 7 days.

**No bladder pain**										**Worst possible bladder pain**
0	1	2	3	4	5	6	7	8	9	10

E45. Please indicate whether you have (had) the following other types of pain **in the last 12 months**:

Low back pain	☐ No	☐ Yes, in last month	☐ Yes, more than 1 month ago
Back pain that goes away with exercise	☐ No	☐ Yes, in last month	☐ Yes, more than 1 month ago
Back pain that does not go away with exercise	☐ No	☐ Yes, in last month	☐ Yes, more than 1 month ago
Muscle/joint pain unrelated to a viral infection or (sports) injury	☐ No	☐ Yes, in last month	☐ Yes, more than 1 month ago
Pain at ovulation (mid cycle)	☐ No	☐ Yes, in last month	☐ Yes, more than 1 month ago
Pain in legs	☐ No	☐ Yes, in last month	☐ Yes, more than 1 month ago
Pain with urination	☐ No	☐ Yes, in last month	☐ Yes, more than 1 month ago
Pain with bowel movement	☐ No	☐ Yes, in last month	☐ Yes, more than 1 month ago

E46. Have you ever been significantly bothered by recurrent headaches?

☐ No → Please skip to question **E59**

☐ Yes → Please continue with question **E47**

E47. Do you still have recurrent headaches?

☐ No ☐ Yes

E48. Have you had at least five separate attacks of headache severe enough to require that you stop or decrease your activities or take a medication for pain?

☐ No → Please skip to question **E59**

☐ Yes → Please continue with question **E49**

E49. Do you have (head) pain-free intervals of days to weeks between severe headache attacks?

☐ No ☐ Yes

E50. If left untreated, would your headache attacks usually last more than four hours and less than three days?

☐ No → Please skip to question **E59**

☐ Yes → Please continue with question **E51**

E51. Are your most troublesome headaches…

(a) Often pulsating (“throbbing”)?

☐ No ☐ Yes

(b) Often unilateral (left or right side of head) for at least a portion of the headache attack?

☐ No ☐ Yes

(c) Severe enough to cause you stop or decrease your activities?

☐ No ☐ Yes

(d) Made worse by physical activity?

☐ No ☐ Yes

E52. Are your headache attacks usually accompanied by ...

(e) Nausea or vomiting?

☐ No ☐ Yes

(f) Sensitivity to light?

☐ No ☐ Yes

(g) Sensitivity to noise?

☐ No ☐ Yes

E53. With at least two of your headache attacks have you had temporary visual disturbances (e.g. shimmering lights, zigzags, blind spots, circles, crescent shapes) just before or during the headache?

☐ No → Please skip to question **E57**
☐ Yes → Please continue with question **E54**

E54. Which of the following **best** describes your visual disturbances? Please choose **only one**.

☐ Light objects appearing excessively bright
☐ All objects appearing grey or yellow
☐ Distortion of all linear objects
☐ Dancing and moving cobwebs
☐ Scintillating picket fences
☐ Zigzag streaks of light
☐ White lights
☐ Heat waves
☐ Sparklers
☐ Silver streaks
☐ Blind spot
☐ None of these (*Please describe):* _______
☐ Flashing gold lights
☐ Moving black veils
☐ Herringbone
☐ Double vision
☐ Silver stars

E55. Does the visual disturbance change (e.g. worsen, change character) within four minutes?

☐ No ☐ Yes

E56. Does the visual disturbance go away completely within 60 minutes?

☐ No ☐ Yes

E57. With at least two of your headache attacks have you had temporary numbness, tingling, or both, involving the lips, tongue, fingers or legs occurring just before or during the headache?

☐ No ☐ Yes

E58. Have you had headaches accompanied by both visual disturbance and temporary numbness/tingling?

☐ No ☐ Yes

E59. Have you ever had any other type of chronic pain lasting at least six months?

☐ No ☐ Yes → **If yes: E59.1.** Which type of pain have you had, for how long (*either continuously or on and off*), and how severe was the pain typically?

**Type of Pain**	**Duration**			**Typical Severity**		
	Less than 1 year	1-5 years	More than 5	Mild	Moderate	Severe
Back Pain						
Neck Pain						
Post-Surgical Pain(including scar pain)						
Other (*Please specify*):						

Medical history

F1. Please tick whether you have had any of the following medical conditions and at what age you were first diagnosed by a doctor. (*Please tick ✓ all that apply*)

Yes	Medical Condition	Age diagnosed	Yes	Medical Condition	Age diagnosed
☐	Anxiety requiring medication or therapy	…….	☐	SLE (Lupus)	…….
☐	Asthma	…….	☐	Migraine	…….
☐	Cardiovascular disease	…….	☐	Pelvic Inflammatory Disease (PID)	…….
☐	Mitral valve prolapse (Murmur syndrome)	…….	☐	Painful bladder/interstitial cystitis (NOT bacterial bladder infection)	…….
☐	Angina	…….	☐	Fibromyalgia	…….
☐	Stroke (Heart attack)	…….	☐	Fibroid uterus	…….
☐	Arrythmias	…….	☐	Glandular fever	…….
☐	Congenital heart disease	…….	☐	Cushing's Disease	…….
☐	Chronic Fatigue Syndrome (CFS) / Myalgic encephalomyelitis (ME)	…….	☐	Crohn's Disease	…….
☐	Deafness/difficulty hearing	…….	☐	Scoliosis (curvature of the spine)	…….
☐	Depression requiring medication or therapy	…….	☐	Spine problems (excluding scoliosis)	…….
☐	Type 1 Diabetes	…….	☐	Sjogren's syndrome	…….
☐	Type 2 Diabetes:<br>☐ Diabetes requiring diet control<br>☐ Diabetes requiring insulin or tablets<br>☐ Diabetes requiring diet, insulin and tablets	…….	☐	Adrenal insufficiency	…….
☐	Insulin resistance	…….	☐	Inflammatory bowel disease (IBD)	…….
☐	Hypoglycemia	…….	☐	Addison's Disease	…….
☐	High cholesterol	…….	☐	Ulcerative Colitis	…….
☐	Thyroid diseases:<br>☐ Hyperthyroid<br>☐ Graves' disease<br>☐ Hypothyroid<br>☐ Hashimoto's Disease<br>☐ Guatr	…….	☐	Fibrosis:<br>☐ Hepatit Fibrosis (Liver)<br>☐ Pulmonary Fibrosis (Lungs)	…….
☐	Vitamin D deficiency	…….	☐	Dupuytren's disease	…….
☐	High blood pressure	…….	☐	Parkinson's disease	…….
☐	Low blood pressure	…….	☐	Multiple Sclerosis (MS)	…….
☐	Uterine Fibroids	…….	☐	Uveitis	…….
☐	Ovarian Cysts	…….	☐	Psoriasis	…….
☐	Eating disorders<br>☐ Bulimia<br>☐ Anorexia<br>☐ Undefined	…….	☐	Thalassamia ☐ Carrier ☐ Diseased<br>Beta Thalassamia ☐ Carrier ☐ Diseased<br>Alpha-thalassamia ☐ Carrier ☐ Diseased	…….
☐	Clinical obesity	…….	☐	Hemochromatosis	…….
☐	Clinical Iron-deficiency	…….	☐	Behcet's disease	…….
☐	Endometriosis (Chocolate cyst)	…….	☐	Eczema	…….
☐	Polycystic Ovary Syndrome (PCOS)	…….	☐	Sickle-cell anemia ☐ Carrier ☐ Diseased	…….
☐	Bone disorders<br>☐ Osteoporosis<br>☐ Rheumatoid Arthritis<br>☐ Reiter's Syndrome (Reactive arthritis)	…….	☐	Early onset dementia: ☐ Alzheimer's<br>☐ Vascular ☐ Frontotemporal<br>☐ Other _________	…….
☐	Irritable Bowel Syndrome (IBS)	…….	☐	Hirsuitism (Male-pattern body hair)	…….
☐	Other: ________________	…….	☐	Other: ________________	…….
☐	Other:________________	…….	☐	Other: ________________	…….

F2. Have you been told that you were born with a structural problem / birth defect of your uterus, cervix, or vagina?

☐ No
☐ Yes → **If yes: F2.1.** Did you have surgery for this issue?
☐ No
☐ Yes → **If Yes: F2.1.1** Was the problem improved or corrected after surgery?
☐ No
☐ Yes

F3. Have you ever been diagnosed by a doctor with cancer or a malignancy of any kind?

☐ No ☐ Yes

If Yes: F3.1. What type(s) of cancer (primary location) have you been diagnosed with, and when were you first diagnosed? *(Please write below)*

**Type of Cancer**	**Age first diagnosed (years)**

F4. Have you had any of the following surgical procedures during your life? If so, at approximately what age(s) did you have the procedure(s), how many have you had in total, and what was the reason for the surgery?

**Surgical Procedures**	**No**	**Yes**	**If Yes:** How many times?	Please list age(s)	What was the reason for the surgery?	**How was this funded?**
Tubal ligation (sterilisation/tubes tied)	☐	☐				☐ State Insurance<br>☐ Private Insurance<br>☐ Out of Pocket
Appendix removed	☐	☐				☐ State Insurance<br>☐ Private Insurance<br>☐ Out of Pocket
Hysterectomy	☐	☐			………………………………<br>………………………………	☐ State Insurance<br>☐ Private Insurance<br>☐ Out of Pocket
Oophorectomy **If yes**, how many of your ovaries have been removed?<br>☐ 1 ☐ both ☐ unsure	☐	☐			………………………………<br>………………………………	☐ State Insurance<br>☐ Private Insurance<br>☐ Out of Pocket
Dilatation and Curettage (D&C)	☐	☐			………………………………<br>………………………………	☐ State Insurance<br>☐ Private Insurance<br>☐ Out of Pocket
Cervical surgery (LEEP or conization)	☐	☐			………………………………<br>………………………………	☐ State Insurance<br>☐ Private Insurance<br>☐ Out of Pocket
Hysteroscopy	☐	☐			………………………………<br>………………………………	☐ State Insurance<br>☐ Private Insurance<br>☐ Out of Pocket
Gall bladder surgery	☐	☐			………………………………<br>………………………………	☐ State Insurance<br>☐ Private Insurance<br>☐ Out of Pocket
Hernia operation	☐	☐			………………………………<br>………………………………	☐ State Insurance<br>☐ Private Insurance<br>☐ Out of Pocket

Surgical Procedures	No	Yes	If Yes: How many times?	Please list age(s)	What was the reason for the surgery?	How was this funded?
Sigmoidoscopy/colonoscopy (insertion of a tube to look inside your bowel)	☐	☐			……………………………………<br>……………………………………	☐ State Insurance<br>☐ Private Insurance<br>☐ Out of Pocket
Laparoscopy (surgery involving insertion of a telescope into you abdomen)	☐	☐			……………………………………<br>……………………………………	☐ State Insurance<br>☐ Private Insurance<br>☐ Out of Pocket
Thyroidectomy (Thyroid removal; total or partial)	☐	☐			……………………………………<br>……………………………………	☐ State Insurance<br>☐ Private Insurance<br>☐ Out of Pocket
Bariatric surgery	☐	☐			……………………………………<br>……………………………………	☐ State Insurance<br>☐ Private Insurance<br>☐ Out of Pocket
Angioplasty	☐	☐			……………………………………<br>……………………………………	☐ State Insurance<br>☐ Private Insurance<br>☐ Out of Pocket
Bypass surgery	☐	☐			……………………………………<br>……………………………………	☐ State Insurance<br>☐ Private Insurance<br>☐ Out of Pocket
Other surgery:<br>………………………………………………….	☐	☐			……………………………………<br>……………………………………	☐ State Insurance<br>☐ Private Insurance<br>☐ Out of Pocket

F5. Have you experienced unusual or excessive hair growth on your face or body that is not a side effect of a medication?

☐ No ☐ Yes

If Yes: F5.1. Where have you experienced such hair growth? On your:

**Upper lip**	☐ Yes	☐ No	**Thighs**	☐ Yes	☐ No
**Chin**	☐ Yes	☐ No	**Chest**	☐ Yes	☐ No
**Upper back**	☐ Yes	☐ No	**Upper abdomen**	☐ Yes	☐ No
**Lower back**	☐ Yes	☐ No	**Lower abdomen**	☐ Yes	☐ No
**Upper arms**	☐ Yes	☐ No			

F6. Have you had acne after the age of 18 (adult acne)?

☐ No ☐ Yes

F6.1. Have you had acne after the age of 25 (adult acne)?

☐ No ☐ Yes

F6.1.1. Where have you had acne? *(Please respond for each item)*

**Face or throat**	☐ Yes	☐ No
**Chest or back**	☐ Yes	☐ No
**Upper arms**	☐ Yes	☐ No

F7. Have you experienced thinning or loss of your hair (not including hair-thinning side-effects of medications or treatments)?

☐ No ☐ Yes

F8. Do you have darkening of the skin around your neck, under your arms, on your hands or in your groin?

☐ No ☐ Yes

F9. The following questions are about your bowel movements/stool in general **in the last 3 months:**

In the last 3 months, how often...	**Never/ Rarely**	**Some-times**	**Often**	**Most of the time**	**Always**
…did you have loose, mushy, or watery stools?	☐	☐	☐	☐	☐
… did you have blood in stools?	☐	☐	☐	☐	☐
…did you have hard or lumpy stools?	☐	☐	☐	☐	☐

F10. Have you had any of the following in the **last month**? (*Please tick ✓all that apply*)

☐ Rectal bleeding or blood in your stool
☐ Less than 3 bowel movements per week
☐ More than 3 bowel movements per day
☐ Passing mucus at the time of bowel movements
☐ Nausea and/or vomiting
☐ Straining during a bowel movement
☐ Urgent need to have a bowel movement
☐ Feeling of incomplete emptying with bowel movements
☐ Abdominal fullness, bloating, or swelling
☐ Intestinal cramping

F11. In the **last 3 months**, have you experienced any of the following? (*Please tick ✓all that apply*)

☐ Loss of urine when coughing, sneezing or laughing
☐ Difficulty passing urine
☐ Frequent bladder infections
☐ Blood in the urine
☐ Still feeling full after urination
☐ Having to urinate again within minutes of urinating

F12. Has a doctor or other health care provider ever diagnosed you with endometriosis?

☐ No → Skip to **F13.**
☐ Yes → **If Yes:**

F12.1. How was the diagnosis made? (*Please tick ✓all that apply*)

☐ laparoscopy or other surgical procedure
☐ ultrasound/MRI scan
☐ based on symptoms
☐ other, please describe: ____

F12.2. If you have had surgery for endometriosis, during your most recent surgery was your endometriosis treated (i.e. was it removed or burnt away)?

☐ No
☐ Yes
☐ Unsure
☐ Have not had surgery for endometriosis

F12.3. How old were you when you first had symptoms? _____ years old

F12.4. What symptoms, if any, prompted you to see a health care provider before your diagnosis with endometriosis? (*Please tick ✓all that apply*)

☐ Pain
☐ Infertility
☐ No symptoms
☐ Other (please specify): ____

F12.5. How old were you when you were diagnosed with endometriosis? _____ years old

F13. Have you ever had surgery to look for endometriosis and none was found?

☐ No

☐ **Yes → If yes: F13.1.** What symptoms prompted the surgery? (*Please tick ✓ all that apply*)

☐ Pain

☐ Infertility

☐ Other *(Please specify):* ____

F14. Have any of your blood relatives been diagnosed with any of the conditions below?
(*Please tick ✓ all that apply)*

**Condition**	**Mother**	**Father**	**Sister**	**Brother**	**Daughter**	**Son**	**Grandparents, aunt, uncle, cousin** **Mother's side**	**Father's side**
Endometriosis	☐		☐		☐		☐	☐
Chronic pelvic pain	☐		☐		☐		☐	☐
Polycystic Ovary Syndrome (PCOS)	☐		☐		☐		☐	☐
Uterine Fibroids	☐		☐		☐		☐	☐
Heavy vaginal bleeding	☐		☐		☐		☐	☐
Thyroid disease	☐	☐	☐	☐	☐	☐	☐	☐
Cardiovascular disease	☐	☐	☐	☐	☐	☐	☐	☐
Diabetes	☐	☐	☐	☐	☐	☐	☐	☐
Dementia	☐	☐	☐	☐	☐	☐	☐	☐
Clinical obesity	☐	☐	☐	☐	☐	☐	☐	☐
Cancer	☐	☐	☐	☐	☐	☐	☐	☐

F15. At what age did your biological mother reach the menopause (stop having periods) naturally?

.... years or: ☐ She has not reached the menopause yet, aged .... years
☐ Don't know / her periods did not stop naturally

F16. At what age did your biological sister(s) reach the menopause (stop having periods) naturally?

……………….years or ☐ She has not reached the menopause yet, aged .... years
☐ Don't know / her periods did not stop naturally

………………years or ☐ She has not reached the menopause yet, aged .... years
☐ Don't know / her periods did not stop naturally

………………..years or ☐ She has not reached the menopause yet, aged .... years
☐ Don't know / her periods did not stop naturally

Medication history

G1. Please tell us about any pain medications, over-the-counter or prescription, that you have used at least once a week for a period of **3 months or longer**.

☐ Yes → Please fill out the **Prescription Drug Table below** **(G1.1)**

☐ No → Skip to **G2.**

G1.1 PAIN RELIEF DRUG TABLE

**Type of drug**	**Ever used?** ✓ *if yes*	**Currently taking?** ✓ *if yes*	**If you are currently taking this drug, how is this funded?**	**At what age did you first take this drug regularly?**	**For what pain was this medication used?**	**How many days per week?**	**How many tablets per week?**	**In total, how long have you used this drug?**
Paracetamol/ acetaminophen Other painkillers: Please specify ______________________	☐	☐	☐ State Insurance ☐ Private Insurance ☐ Out of Pocket	____	☐ Pelvic pain ☐ Other pain ☐ Both	☐ 1 ☐ 2-3 ☐ 4-5 ☐ 6+	☐ 1-2 ☐ 3-5 ☐ 6-14 ☐ 15+	____ months ____ years
Aspirin (325 mg or more/tablet)	☐	☐	☐ State Insurance ☐ Private Insurance ☐ Out of Pocket	____	☐ Pelvic pain ☐ Other pain ☐ Both	☐ 1 ☐ 2-3 ☐ 4-5 ☐ 6+	☐ 1-2 ☐ 3-5 ☐ 6-14 ☐ 15+	____ months ____ years
Ibuprofen (e.g., Brufen,Nurofen)	☐	☐	☐ State Insurance ☐ Private Insurance ☐ Out of Pocket	____	☐ Pelvic pain ☐ Other pain ☐ Both	☐ 1 ☐ 2-3 ☐ 4-5 ☐ 6+	☐ 1-2 ☐ 3-5 ☐ 6-14 ☐ 15+	____ months ____ years
Celebrex, Vioxx (COX-2 inhibitors)	☐	☐	☐ State Insurance ☐ Private Insurance ☐ Out of Pocket	____	☐ Pelvic pain ☐ Other pain ☐ Both	☐ 1 ☐ 2-3 ☐ 4-5 ☐ 6+	☐ 1-2 ☐ 3-5 ☐ 6-14 ☐ 15+	____ months ____ years
Other anti-inflammatory analgesics (naproxen, mefanamic acid, Aleve, Naprosyn, Relafen, Ketoprofen, Anaprox)	☐	☐	☐ State Insurance ☐ Private Insurance ☐ Out of Pocket	____	☐ Pelvic pain ☐ Other pain ☐ Both	☐ 1 ☐ 2-3 ☐ 4-5 ☐ 6+	☐ 1-2 ☐ 3-5 ☐ 6-14 ☐ 15+	____ months ____ years
Strong (narcotic) analgesics (hydrocodone +paracetamol, codeine+paracetamol, morphine, codeine, oxycodone, hydrocodone, Demerol)	☐	☐	☐ State Insurance ☐ Private Insurance ☐ Out of Pocket	____	☐ Pelvic pain ☐ Other pain ☐ Both	☐ 1 ☐ 2-3 ☐ 4-5 ☐ 6+	☐ 1-2 ☐ 3-5 ☐ 6-14 ☐ 15+	____ months ____ years
Other pain-killing drugs aimed at the nerves/central nervous system (amitriptyline, nortryptilline, gabapentin, pregabalin, lamotrogine)	☐	☐	☐ State Insurance ☐ Private Insurance ☐ Out of Pocket	____	☐ Pelvic pain ☐ Other pain ☐ Both	☐ 1 ☐ 2-3 ☐ 4-5 ☐ 6+	☐ 1-2 ☐ 3-5 ☐ 6-14 ☐ 15+	____ months ____ years
Muscle relaxants (diazepam/temazepam, buscopan)	☐	☐	☐ State Insurance ☐ Private Insurance ☐ Out of Pocket	____	☐ Pelvic pain ☐ Other pain ☐ Both	☐ 1 ☐ 2-3 ☐ 4-5 ☐ 6+	☐ 1-2 ☐ 3-5 ☐ 6-14 ☐ 15+	____ months ____ years
Herbal medicines (e.g. Capsicum,Vitex agnus-cactus)	☐	☐	☐ State Insurance ☐ Private Insurance ☐ Out of Pocket	____	☐ Pelvic pain ☐ Other pain ☐ Both	☐ 1 ☐ 2-3 ☐ 4-5 ☐ 6+	☐ 1-2 ☐ 3-5 ☐ 6-14 ☐ 15+	____ months ____ years

G2. Have you EVER taken prescription drugs for more than 3 months, excluding hormone treatments and pain medications?

☐ Yes → Please fill out the **Prescription Drug Table below (G2.1)**
☐ No → Please skip to **H1**

G2.1. PRESCRIPTION DRUG TABLE

**Type of drug**	**Have you ever taken this drug every day for over a month?**	**At what age did you first take this drug every day for over a month?**	**In total, how many years you have taken this drug? Please estimate, and enter "0 total years" if less than 1 year.**	**Are you currently taking this drug every day?**	**If you are currently taking this drug, how is this funded?**	**Please write down the specific name of the drug you have used most recently if known:**
	✓ *if yes*	*Age 1st*	*Years taken:*	✓ *if yes*		*Name of drug:*
Diuretic (water pill)	☐	__ __	__ __	☐	☐ State Insurance<br>☐ Private Insurance<br>☐ Out of Pocket	
Diabetic tablets (e.g. metformin)	☐	__ __	__ __	☐	☐ State Insurance<br>☐ Private Insurance<br>☐ Out of Pocket	
Insulin	☐	__ __	__ __	☐	☐ State Insurance<br>☐ Private Insurance<br>☐ Out of Pocket	
Thyroid drugs (e.g. Levothyrox)	☐	__ __	__ __	☐	☐ State Insurance<br>☐ Private Insurance<br>☐ Out of Pocket	
Drugs for epilepsy	☐	__ __	__ __	☐	☐ State Insurance<br>☐ Private Insurance<br>☐ Out of Pocket	
Sleeping tablets / tranquilisers	☐	__ __	__ __	☐	☐ State Insurance<br>☐ Private Insurance<br>☐ Out of Pocket	
Anti-depressants	☐	__ __	__ __	☐	☐ State Insurance<br>☐ Private Insurance<br>☐ Out of Pocket	
Other drugs to treat mental illness	☐	__ __	__ __	☐	☐ State Insurance<br>☐ Private Insurance<br>☐ Out of Pocket	
Drugs for osteoporosis ("brittle bones")	☐	__ __	__ __	☐	☐ State Insurance<br>☐ Private Insurance<br>☐ Out of Pocket	
Drugs for rheumatoid arthritis	☐	__ __	__ __	☐	☐ State Insurance<br>☐ Private Insurance<br>☐ Out of Pocket	
Antibiotics for a month or more	☐	__ __	__ __	☐	☐ State Insurance<br>☐ Private Insurance<br>☐ Out of Pocket	
Antacids	☐	__ __	__ __	☐	☐ State Insurance<br>☐ Private Insurance<br>☐ Out of Pocket	
Drugs for stomach ulcer / gastritis	☐	__ __	__ __	☐	☐ State Insurance<br>☐ Private Insurance<br>☐ Out of Pocket	

Type of drug	Have you ever taken this drug every day for over a month?	At what age did you first take this drug every day for over a month?	In total, how many years you have taken this drug? Please estimate, and enter "0 total years" if less than 1 year.	Are you currently taking this drug every day?	If you are currently taking this drug, how is this funded?	Please write down the specific name of the drug you have used most recently if known:
	✓ *if yes*	*Age 1st*	*Years taken:*	✓ *if yes*		*Name of drug:*
Drugs for high cholesterol (e.g. Statins)	☐	__ __	__ __	☐	☐ State Insurance<br>☐ Private Insurance<br>☐ Out of Pocket	
Drugs for allergies (antihistamines)	☐	__ __	__ __	☐	☐ State Insurance<br>☐ Private Insurance<br>☐ Out of Pocket	
Steroids (oral, inhaled, or nasal)	☐	__ __	__ __	☐	☐ State Insurance<br>☐ Private Insurance<br>☐ Out of Pocket	
Chemotherapy for cancer	☐	__ __	__ __	☐	☐ State Insurance<br>☐ Private Insurance<br>☐ Out of Pocket	
Tamoxifen for cancer	☐	__ __	__ __	☐	☐ State Insurance<br>☐ Private Insurance<br>☐ Out of Pocket	
Drugs for Blood pressure (e.g. beta-blockers)	☐	__ __	__ __	☐	☐ State Insurance<br>☐ Private Insurance<br>☐ Out of Pocket	
Drugs for angina (chest pain) (e.g. beta-blockers)	☐	__ __	__ __	☐	☐ State Insurance<br>☐ Private Insurance<br>☐ Out of Pocket	
Other drugs for a heart condition (e.g. beta-blockers)	☐	__ __	__ __	☐	☐ State Insurance<br>☐ Private Insurance<br>☐ Out of Pocket	
Inhaler for asthma	☐	__ __	__ __	☐	☐ State Insurance<br>☐ Private Insurance<br>☐ Out of Pocket	
Warfarin / heparin to thin blood	☐	__ __	__ __	☐	☐ State Insurance<br>☐ Private Insurance<br>☐ Out of Pocket	
Migraine tablets/injections	☐	__ __	__ __	☐	☐ State Insurance<br>☐ Private Insurance<br>☐ Out of Pocket	
Vitamin D supplements	☐	__ __	__ __	☐	☐ State Insurance<br>☐ Private Insurance<br>☐ Out of Pocket	
Other 1: ……………………………..	☐	__ __	__ __	☐	☐ State Insurance<br>☐ Private Insurance<br>☐ Out of Pocket	
Other 2: ……………………………..	☐	__ __	__ __	☐	☐ State Insurance<br>☐ Private Insurance<br>☐ Out of Pocket	
Other 3: ……………………………..	☐	__ __	__ __	☐	☐ State Insurance<br>☐ Private Insurance<br>☐ Out of Pocket	

Personal Information and lifestyle

H1. What is your date of birth? __ __ / __ __ / __ __ __ __
DD MM YYYY

H2. How would you describe your ethnic origin?

☐ Cypriot → ☐ Turkish Cypriot ☐ Greek Cypriot ☐ Maronite ☐ Armenian ☐ Latin

☐ Turkish
☐ Greek
☐ Mixed: ______________________________________ *(Please specify)*
☐ Other: ______________________________________ *(Please specify)*

H3. What is your place of birth? (City/Country) __________________________

H4. Was your mother or father born in Cyprus?

☐ No
☐ Yes
☐ Do not know

If no → **H4.1.** Your father's place of birth (City/Country): ____________________ ☐Do not know

H4.2. Your mother's place of birth (City/Country):____________________ ☐Do not know

H5. Was one of your grandparents born outside Cyprus?

☐ No
☐ Yes → **If yes, please specify: ________________________**
☐ Do not know

H6. What is your marital status?

☐ Single (never married) ☐ Married ☐ Divorced/ separated ☐ Widowed

H7. Are you currently living with a partner?

☐ No, I am not in a relationship ☐ No, but I am in a relationship ☐ Yes

H8. Are you currently in school?

☐ No ☐ Yes

H9. What is the highest level of education you have attained (with certificate)?

☐ Primary school ☐ Post-secondary not university / some college or vocational school
☐ Middle school ☐ University
☐ High school ☐ Postgraduate

H10. What is your occupation/job? _________________________________

H11. What is your current height? _______ cm or if you prefer, ______ feet ______ inches

H12. What is your current weight? _______ kg or if you prefer, _______ pounds or ______ stones

H13. Since 18 years of age, what is **the most** that you have weighed (*Not including pregnancy and the 12 months following pregnancy*)?_______ kg or if you prefer, _______ pounds or ______ stones

H13.1. How old were you when you weighed that amount? _____

H14. Since 18 years of age, what is **the least** that you have weighed? _______ kg or if you prefer, _______ pounds or ______ stones

H14.1. How old were you when you weighed that amount? ______

H15. Do you know what your weight/height at birth was?

☐ No ☐ Yes

If Yes: H15.1.: Birth weight: ……….pounds ……….ounces or ……………….kilograms

H15.2.: Birth height: ………… cm or …………… inches

H16. At age 18, what was your natural hair colour? *(Please tick one)*

☐ Red ☐ Dark brown ☐ Blonde ☐ Light brown ☐ Black

H17. What is your eye colour? *(Please tick one)*

☐ Blue ☐ Hazel ☐ Gray ☐ Brown ☐ Green

H18. How would you describe your natural skin tone before tanning or on areas not exposed to the sun (*e.g. on your upper inner arm*)?

☐ Very light or white (often sunburns)
☐ Light (usually sunburns)
☐ Light intermediate (rarely sunburns)
☐ Dark intermediate (rarely sunburns)
☐ Dark (sunburns very rarely)
☐ Very dark (sunburns extremely rarely)

H19. Moles are brown or black spots on the skin, which usually start in childhood. They may be flat (cannot be felt) or raised (can be felt). Moles are usually darker and larger than freckles. Moles usually appear on their own, whereas freckles appear in groups. A spot that looks like a freckle but is on its own and cannot be felt is most likely a mole.

Using the diagrams below, which picture best describes how many moles you have on your body *(Please select one picture)*?

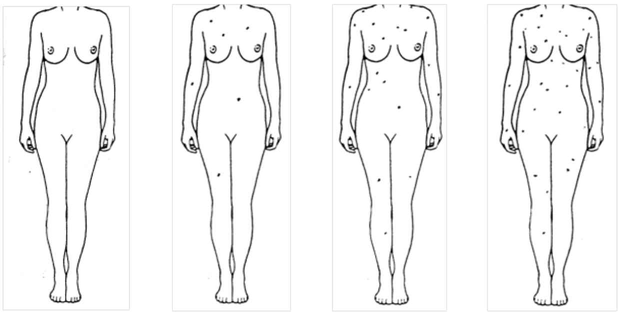

☐ No moles ☐ 1-10 moles ☐ 11-50 moles ☐ 50 and more moles

H20. On average, how much time in a week do you spend under sunlight (job, leisure, gardening, sports etc.) during the day (between 10a.m. and 4p.m.)?

	Less than an hour per week	2-4 hours a week	5+ hours a week
**During Summer (April-October)**			
Age <25	☐	☐	☐
Between ages 26–35	☐	☐	☐
Between ages 36–45	☐	☐	☐
Between ages 46–55	☐	☐	☐
In last 2 years	☐	☐	☐
**During Winter (November-March)**			
Ages <25	☐	☐	☐
Between ages 26–35	☐	☐	☐
Between ages 36–45	☐	☐	☐
Between ages 46–55	☐	☐	☐
In last 2 years	☐	☐	☐

H21. Do you regularly use sunscreen with more than 15 spf?

During summer: ☐Yes ☐No
During Winter: ☐Yes ☐No

H22. How many times **in a year** do you use solarium? (Please specify for the given time periods)

	None	1-2 times	3-5 times	6-11 times	12-23 times	24+ times
Summer months: Age <25	☐	☐	☐	☐	☐	☐
In summer between ages 26-34	☐	☐	☐	☐	☐	☐
In summer between ages 36-45	☐	☐	☐	☐	☐	☐
In summer between ages 46-55	☐	☐	☐	☐	☐	☐
In last 2 years	☐	☐	☐	☐	☐	☐

H23. During the **last 12 months**, what was your average time per week spent on each of the following recreational activities?

	Zero	1-4 min	5-19 min	20-59 min	One hour	1-1.5 hours	2-3 hours	4-6 hours	7-10 hours	11+ hours
Walking or hiking outdoors (include walking to work)	☐	☐	☐	☐	☐	☐	☐	☐	☐	☐
Jogging (slower than 10 minutes/mile)	☐	☐	☐	☐	☐	☐	☐	☐	☐	☐
Running (10 minutes/mile or faster)	☐	☐	☐	☐	☐	☐	☐	☐	☐	☐
Bicycling (include stationary machine)	☐	☐	☐	☐	☐	☐	☐	☐	☐	☐
Calisthenics/aerobics/aerobic dance/rowing machine	☐	☐	☐	☐	☐	☐	☐	☐	☐	☐
Tennis, squash, racquetball	☐	☐	☐	☐	☐	☐	☐	☐	☐	☐
Lap swimming	☐	☐	☐	☐	☐	☐	☐	☐	☐	☐
Other aerobic recreation (e.g., lawn mowing)	☐	☐	☐	☐	☐	☐	☐	☐	☐	☐

H24. Have you smoked more than 100 cigarettes during your lifetime?

☐ No ☐ Yes

If yes: **H24.1.** How old were you when you first started smoking? _______ years old

H24.2. Do you smoke currently?

☐ No, I stopped smoking at age ________

☐ Yes, and I smoke about ________ cigarettes per week

H25. Do you drink any alcohol?

☐ No ☐ Yes

If yes: During an average week, how much do you drink of each of the following?

(*Please note exact numbers, not ranges such as 1-3*)

**Type of alcohol (serving size)**	**Average number of each drink per week**
Beer/lager/cider (330ml)	....
Whisky (50 ml)	....
Wine (125 ml)	....
Spirits, e.g. vodka (100 ml)	....
Raki (85ml)	....
Shots, e.g. zivania (15ml)	....
Other (*Please specify*)	....

H26. What term best describes your current work status?

☐ Working in a paid job, as an employee

☐ Self-employed

☐ Not in paid work force: *(Please tick ✓ all that apply)*

- ☐ Homemaker
- ☐ Unable to work because of the symptoms for which I am undergoing surgery
- ☐ In full time education
- ☐ Unable to work for other reasons
- ☐ Doing voluntary work
- ☐ Other (*Please describe*)…………………………………….

H27. **During the past four weeks**, how many days or hours did you miss from work because of problems associated with your symptoms? Include hours you missed on sick days, times you went in late, left early, etc., because of problems associated with your symptoms.

……….. days   or ………..hours

H28. During the past four weeks, how many days or hours did you miss from work because of any other reason, such as vacation, holidays, time off to participate in this study?

………. days   or ………. hours

H29. During the past four weeks, how many days or hours did you actually work?

……….. days   or ………. hours

H30. During the past four weeks, how much did your symptoms affect your productivity **while you were working**? *Think about days you were limited in the amount or kind of work you could do, days you accomplished less than you would like, or days you could not do your work as carefully as usual. If your symptoms affected your work only a little, choose a low number. Choose a high number if your symptoms affected your work a great deal.*

You are asked about overall productivity on days you actually went to work. If productivity differed greatly from day to day, for example one day was 0 and one day was 10, please respond for all days, on average.

Consider only how much your symptoms affected productivity while you were working

Symptoms had no effect on my work — **Symptoms completely prevented me from working**

0 1 2 3 4 5 6 7 8 9 10

H31. During the past four weeks, how much did your symptoms affect your ability to do your regular daily activities, **other than work at a job**? *By regular activities, we mean the usual activities you do, such as work around the house, shopping, childcare, exercising, studying, etc. Think about times you were limited in the amount or kind of activities you could do and times you accomplished less than you would like. If your symptoms affected your activities only a little, choose a low number. Choose a high number if you symptoms affected your activities a great deal.*

You are asked about overall effect on your activities. If the effect differed greatly from day to day, for example one day was 0 and one day was 10, please respond for all days, on average.

Consider only how much your symptoms affected your ability to do your regular activities, other than work at a job.

Symptoms had no effect on my daily activities — **Symptoms completely prevented me from doing my daily activities**

0 1 2 3 4 5 6 7 8 9 10

H32. How many hours a week do you get paid to work? (If self-employed, specify the number of hours a week that your work on average): ………………hours

Thank you for your time and cooperation in answering these questions. If you have comments or questions about any part of this survey, please explain here.

____

____

____

____

____
