# Supplementary material for: Cyprus Women’s Health Research (COHERE) initiative: determining the relative burden of women’s health conditions and related co-morbidities in an Eastern Mediterranean population
Source: BMC Womens Health. 2019 Apr 3;19:50. doi: 10.1186/s12905-019-0750-1 (PMC6446287; doi:10.1186/s12905-019-0750-1)
Supplement: Supplementary file 2 — Power calculations. (DOCX 15 kb) [file 12905_2019_750_MOESM2_ESM.docx]

**Power Calculations**

We are aiming to recruit a random ~10% sample of 82,104 women between ages of 18-55. Larger sample sizes allow better representation of rural, less populated regions as well as less frequently seen or diagnosed conditions. This is a nationwide study and we aim to identify disease with prevalence rates as low as 0.5%. Moreover, where we have the needed case numbers we aim to look for association between lifestyle factors and enrichment of related conditions within families. Here are the conducted power calculations:

We used the following formula^[1]^ for sample size calculations: *n* = [*Z* ^2^ **P* (1 − *P*)]/*d*^2^ where *n* = sample size, *Z* = Z statistic for a level of confidence, *P* = expected prevalence or proportion, and *d* = precision. For obtaining a 95% confidence interval, we have used the corresponding Z statistic value of 1.96. For the precision level (d), as routinely used in practice and stated by Naing et al ^[2]^, we used 5% level for expected prevalence rates (P) between 10-90%, P divided by 2, for expected prevalence rates between 0-10%, and 1-P divided by 2, for expected prevalence rates between 90-100%. In **Table 1** below, you can see the minimum sample sizes that would be required or sufficient to give us the desired precision for the calculated 95% confidence interval for prevalence. Hence, to cover expected prevalence rates down to 0.05% for all diseases of interest, a minimum sample size of 3058 volunteers would be needed.

**Table 1.** Sample size calculations for estimating prevalence rate with desired precision.

| **Expected Prevalence (P)** | **Desired Precision (d)** | **Attainable 95% Confidence Interval for Prevalence** | **Minimum Sample Size (n)** |
| --- | --- | --- | --- |
| 0.1% | 0.05% | 0.05-0.15% | 15352 |
| 0.5% | 0.25% | 0.25-0.75% | 3058 |
| 1% | 0.5% | 0.5-1.5% | 1522 |
| 2% | 1% | 1-3% | 753 |
| 5% | 2.5% | 2.5-7.5% | 292 |
| 10% | 5% | 5-15% | 139 |
| 25% | 5% | 20-30% | 289 |
| 50% | 5% | 45-55% | 385 |
| 75% | 5% | 70-80% | 289 |
| 90% | 5% | 85-95% | 139 |
| 95% | 2.5% | 92.5-97.5% | 292 |
| 98% | 1% | 97-99% | 753 |
| 99% | 0.5% | 98.5-99.5% | 1522 |

Moreover, as secondary aims, we would also like to explore the association between various symptoms/behaviours and diseases. To be able to power these analyses, we plan to target a total sample size of 8000 women. The minimum detectable differences in the rate of symptoms/ behaviours between cases and non-cases have been summarized in **Table 2** below for the proposed sample size and diseases with varying expected prevalence rates. As shown in Table 2, with a sample size of 8000 volunteers, we will be able to detect, at a minimum, 2-42% increase in exposure rate in cases compared to non-cases for disease prevalence rates of 0.1-50%.

**Table 2:** Minimum increase in the rate of symptom/behavior detectable at an overall alpha level of 0.05 with 80% power between disease cases and non-cases, assuming true exposure rate among non-cases of 10-90% (using 2-sided Fisher’s exact test).

| **Disease Prevalence** | **Sample sizes Cases / Non-cases** | **True exposure rate among non-cases** | | | | |
| --- | --- | --- | --- | --- | --- | --- |
|  |  | **10%** | **25%** | **50%** | **75%** | **90%** |
| 0.1% | **8/7992** | +42% | +49% | +46% | N/A | N/A |
| 0.5% | **40/7960** | +16% | +21% | +23% | +18% | N/A |
| 1% | **80 / 7920** | +11% | +15% | +16% | +13% | +8% |
| 2% | **160 / 7840** | +8% | +10% | +11% | +10% | +6% |
| 5% | **400 / 7600** | +5% | +7% | +7% | +6% | +4% |
| 10% | **800 / 7200** | +3% | +5% | +6% | +5% | +3% |
| 25% | **2000 / 6000** | +2% | +3% | +4% | +3% | +2% |
| 50% | **4000 / 4000** | +2% | +3% | +3% | +3% | +2% |

**References**

[1] Daniel WW. Biostatistics: A Foundation for Analysis in the Health Sciences. 7th edition. 1999 New York: John Wiley & Sons.

[2] Naing L, Winn T and Rusli BN. Sample Size Calculator for Prevalence Studies. 2006. Available at: http://www.kck.usm.my/ppsg/stats_resources.htm
